# TAK1 operates at the primary cilium in non-canonical TGFB/BMP signaling to control heart development

**DOI:** 10.1371/journal.pbio.3003902

**Published:** 2026-08-04

**Authors:** Canan Doganli, Oskar Kaaber Thomsen, Daniel A. Baird, Yeasmeen Ali, Menachem V. K. Sarusie, Enrique Audain, Line Jeanett Jessen, Pauline Munck Truelsen, Johanne Bay Mogensen, Maria Schrøder Holm, Kateřina Apolínová, Lorenzo Buttò, Maria Diamanti, Jindřiška Leischner Fialová, Emma M. Wade, Stephen P. Robertson, Lotte Bang Pedersen, Laurent Argiro, Fabienne Lescroart, Marc-Phillip Hitz, Søren Tvorup Christensen, Lars Allan Larsen

**Affiliations:** 1 Department of Cellular and Molecular Medicine, University of Copenhagen, Copenhagen, Denmark; 2 Department of Biology, University of Copenhagen, Copenhagen, Denmark; 3 Department of Medical Genetics, Carl von Ossietzky University, Oldenburg, Germany; 4 ZeClinics SL, Badalona, Spain; 5 Faculty of Health and Life Sciences, Universitat Pompeu Fabra, Barcelona, Spain; 6 Department of Women’s and Children’s Health, Dunedin School of Medicine, University of Otago, Dunedin, New Zealand; 7 Aix Marseille Université, INSERM, MMG U1251, Marseille, France; 8 College of Health Sciences, VinUniversity, Hanoi, Vietnam; University of Pennsylvania School of Medicine, UNITED STATES OF AMERICA

## Abstract

Pathogenic variants in the genes encoding the non-canonical TGFB signaling components TAK1 (MAP3K7*)*, TAB2 and PKA-Cα (PRKACA) cause rare multisystem disorders, which may include congenital heart disease (CHD). To investigate the role of TAK1 signaling in CHD, we performed genetic analysis of CHD patients and discovered an increased burden of rare *TAB2* and *TAK1* variants in patients with extracardiac abnormalities. To address the mechanism of TAK1 in heart development, we performed experiments in cell and animal models. Zebrafish *tak1* and *tab2* mutants presented with cardiac and extracardiac developmental defects, and *tak1* mutant hearts showed downregulation of genes encoding core cardiac transcription factors, sarcomeric proteins and extracellular matrix proteins. In vitro experiments indicated that TAK1 via TAB2 and PKA-Cα is activated at the primary cilium during cardiomyogenesis; activation at this site is enhanced by TGFB/BMP ligands. Inactivation of TAK1 inhibited ciliary signaling and cardiomyocyte differentiation, and patient-derived TAK1 variants reduced its ciliary localization. In conclusion, our data establish a pivotal role for TAK1 and its upstream regulators at the primary cilium in heart development and syndromic CHD.

## Abstract 2

Pathogenic variants in genes encoding non-canonical TGFB signaling components, such as TAK1, can cause multisystem disorders that include congenital heart disease, but their specific function remain unclear. This study shows that TAK1 signaling is activated at the primary cilium and regulates cardiomyocyte differentiation.

## Introduction

The heart is the first organ to form during human embryonic development, requiring precise spatial and temporal coordination of morphogens, signaling pathways, transcription factors and chromatin modulators to regulate proliferation, differentiation, and positioning of cardiac progenitor cells [1,2]. Disruptions in these tightly regulated processes can result in congenital heart disease (CHD), a broad spectrum of structural malformations of the heart or intra-thoracic vessels that affect cardiac function [3,4]. Despite advancements in genetics and developmental biology, many aspects of CHD pathogenesis remain unresolved, particularly the role of primary cilia in coordinating cardiogenic signaling [5].

Primary cilia are microtubule-based organelles that project from the surface of quiescent cells and facilitate signal exchange with surrounding tissues [6,7]. Studies have linked primary cilia to heart development [5,8], but how primary cilia orchestrate cardiogenic signals remains poorly understood. This knowledge gap presents a significant challenge in deciphering CHD etiology, particularly in cases where CHD presents in isolation (non-syndromic CHD, nsCHD) or alongside extracardiac anomalies (syndromic CHD, sCHD). Understanding the precise molecular mechanisms by which primary cilia mediate heart development is critical for identifying novel therapeutic targets for CHD.

Transforming Growth Factor-Beta-Activated Kinase 1 (TAK1, also known as MAP3K7) is part of the non-canonical branch of TGFB/BMP signaling and is activated by phosphorylation mediated by the catalytic alpha subunit of cAMP-dependent protein kinase (PKA-Cα, encoded by the *PRKACA* gene) and via interaction with TAK1 Binding Protein 2 (TAB2) [9,10]. Rare genomic and intragenic variants, which disrupt the normal function of *TAB2*, have been implicated in TAB2 haploinsufficiency syndrome (THS), characterized by multisystem abnormalities, including CHD [11,12]. Similarly, rare gain- or loss-of-function mutations in *TAK1* and *PRKACA* contribute to frontometaphyseal dysplasia (FMD, OMIM #617137), cardiospondylocarpofacial syndrome (CSCFS, OMIM #157800) [13,14], and cardioacrofacial dysplasia (CAFD, OMIM #619142) [15–17], multisystem disorders that frequently include CHD. In addition, a previous study suggested that TAK1 plays a role in regulating in vitro cardiomyogenesis [18]. However, despite these connections, the mechanistic contributions of TAK1, TAB2, and PKA-Cα to heart development and multisystem disorders, including sCHD, remain largely undefined. Given the frequent association of TAK1, TAB2, and PRKACA variants with cardiac defects, there is an urgent need to elucidate how these proteins regulate heart formation.

Here, we demonstrate that TAK1, TAB2 and PKA-Cα function as integral components of a collaborative signaling axis that operates at the primary cilium to regulate cardiogenesis. Using zebrafish models, we show that zebrafish *tak1* and *tab2* homozygous mutant larvae recapitulate key developmental defects observed in FMD/CSCFS and THS patients, including heart malformations. RNA sequencing of dissected hearts reveals that Tak1 functions upstream of core cardiac transcription factors and plays a role in both extracellular matrix (ECM) and myocardium development. Through complementary analyses in cardiogenic gastruloids and cell model systems, we find that TAK1, in coordination with TAB2 and PKA-Cα, mediates a non-canonical TGFB/BMP signaling pathway at the primary cilium, essential for cardiomyogenesis. Furthermore, perturbations in TAK1 activity disrupt JNK1/2 activation at the cilium, impeding the timely initiation and progression of in vitro cardiomyocyte differentiation. These findings highlight the critical involvement of the TAK1 signaling axis in cilium-mediated cardiogenesis and provide novel insights into how defects in ciliary signaling contribute to sCHD.

## Methods

### Burden of *TAK1*, *TAB2* and *PRKACA* variants in CHD patients

Variant burden was calculated using ES data from 3,876 CHD cases and 45,082 controls [19]. This study was based on written informed consent and conformed to the principles outlined in the Declaration of Helsinki. The study was approved under the ethics approval EA2/131/10 (Berlin, Germany). Control data was accessed via the UK Biobank 50K WES dataset freeze under the application number 44165. Briefly, case and control data were processed and harmonized using the same alignment (BWA v0.7), variant calling (GATK v4.1), variant annotation (VEP API 96) and quality control (Hail v0.2) pipelines. Rare variants (MAF < 0.001) were defined based on their population and cohort-specific allelic frequency. Protein-altering variants (PAVs) include loss-of-function variants and cover the following VEP classifications; ‘missense_variant’, ‘stop_gained’, ‘frameshift_variant’, ‘inframe_deletion’ and ‘splice_region_variant’. The number of individuals with rare (MAF < 0.001, gnomAD v2.1.1 and 3.0.0) synonymous variants (SYNs) and PAVs in *TAK1*, *TAB2* and *PRKACA* was identified for cases and controls. The variant burden was calculated as the fraction of individuals with variants, divided by the total number of individuals in each group. Potentially pathogenic variants were defined as variants with a Combined Annotation Dependent Depletion (CADD) score > 25 [20]. The statistical significance of differences in variant burden was calculated using Fisher’s exact test.

### Zebrafish maintenance

Embryos were maintained and staged as previously described [21] and all experiments were approved and conducted according to licenses (2017-15-0201-01279, 2022-15-0202-00149) approved by Danish Ministry of Food, Agriculture and Fisheries and guidelines from the Danish Animal Experiments Inspectorate under the Directive 2010/63/EU of the European Parliament and of the Council of 22 September 2010 on the protection of animals used for scientific purposes. Zebrafish larvae were anesthetized using 0.016% or 0.005% (for contractility recordings) tricaine methanesulfonate (Sigma, #MS222, tricaine). Zebrafish larvae were euthanized using 0.2% tricaine. When required, pigmentation was inhibited by supplementing E3 medium (5 mM NaCl, 0.33 mM MgSO_4_, 0.33 mM CaCl_2_, 0.17 mM KCl, pH 7.4) with 0.003% 1-phenyl-2-thiourea (PTU) starting at 24 hpf.

### Zebrafish CRISPR mutants

The *tak1* (also called *map3k7*, ENSDARG00000020469) and *tab2* (ENSDARG00000021509) CRISPR mutants were generated by injecting gRNA and Cas9 mRNA into one-cell stage AB wild type (WT) strain zebrafish, acquired from European Zebrafish Resource Center (EZRC). The F0 embryos were raised to adulthood and bred to AB zebrafish line. F1 generation was screened for mutation by colony PCR and Sanger sequencing, and founders were bred to *Tg(myl7:GFP; kdrl:mCherry)* line. All mutant analyses were performed on F3-5 embryos, generated by breeding of heterozygous adults. As controls, either a mix of +/+ and +/− siblings from +/− adult breeding or +/+ embryos from +/+ (siblings of +/− adults) breeding were used. Genotyping of the larvae was done by PCR (primers are listed in S5 Table) and Sanger sequencing.

### Pharmacological inhibition of Tak1 in zebrafish

Inhibitors, Takinib (Sigma, #SML2216, ToCris, #6430) and (5Z)-7-oxozeaenol (5ZO) (ToCris, #3604), were prepared as 60 and 10 mM stock solutions in 100% DMSO, respectively, and freshly diluted in E3 medium to the indicated concentrations. AB WT or *Tg(myl7:GFP)* zebrafish embryos were dechorionated at 24 hpf, transferred to multiwell plates, and exposed to compounds dissolved in the 1% DMSO/E3. Embryos were exposed to 10 μM Takinib or 5 μM 5ZO, each prepared in 1% DMSO/E3. The medium, with or without compounds, was refreshed at 2 dpf and 4 dpf. Control groups received 1% DMSO/E3 as a vehicle control. All embryos were maintained at 28.5 °C for the indicated duration prior to phenotypic analysis.

### Zebrafish immunostaining, cell counts and assessment of cardiomyocyte proliferation index

For wholemount immunostaining, zebrafish larvae were incubated in 0.2% tricaine for 1 min, fixed in 4% paraformaldehyde (PFA) overnight at 4 °C, washed with PBS-T (1× phosphate-buffered saline (PBS) with 0.1% Tween-20), permeabilized with 0.5% Triton X-100 overnight at 4 °C and blocked in 5% BSA and 5% goat serum for 2 h at RT. Primary and secondary antibodies were added overnight at 4 °C, and washed with PBS-T. DAPI staining solution (1 μg/ml) was added for 30 min and larvae were washed with PBS-T prior to imaging. For extracted heart immunostaining, protocol was used as previously described [22]. Hearts were extracted either manually or using a filter method as described in Lombardo *and colleagues* [23]. Zeiss LSM 780 or 980 confocal microscopes were used for imaging. A straight line at the narrowest point of the atrioventricular canal was used as a defining anatomical landmark, facilitating clear distinction between the atrium and the ventricle. For cell size measurements, the area of the cells from the central chamber regions were defined using the selection tool and measured by Fiji [24]. All cell counts were performed using the Cell Counter in Fiji. Cardiomyocyte proliferation index was calculated after counting cells in the extracted hearts immunostained with Mef2a/c and Pcna antibodies, using the formula: (Mef2^+^ Pcna^+^ cells)/(Mef2^+^ cells). For full list of primary and secondary antibodies used for immunostaining, please see S3 and S4 Tables, respectively.

### Zebrafish in situ hybridization

Plasmids for *nkx2.5* and *mef2cb* probes were kind gifts of Chris Derrick from Newcastle University and Yaniv Hinits from King´s College London, respectively. The plasmids were linearized by EcoRI (*nkx2.5* plasmid) and SacII (*mef2cb* plasmid) enzyme (New England Biolabs) and transcribed with T7 RNA polymerase (Roche). The RT-PCR fragment of *elastin b* was amplified (primers listed in S5 Table) and cloned into the pCRII-TOPO vector (Invitrogen) for riboprobe synthesis. The plasmid was linearized by NcoI enzyme (New England Biolabs) and transcribed with SP6 RNA polymerase (Roche). Digoxigenin (DIG)-labeled probes were used for in situ hybridization and detected either with FastRed or NBT/BCIP (Roche). In situ hybridization was performed as described [25]. For *elnb* in situ hybridization, anti-GFP antibody was incubated together with anti-DIG-AP Fab fragments. Following PBST washes, samples were incubated with Alexa Fluor 488-conjugated goat anti-chicken secondary antibody for 2 hours at room temperature prior to development of the FastRed staining. Embryos were mounted ventrally in 100% glycerol and imaged on a Zeiss Axio Zoom.V16 microscope equipped with a PlanNeoFluar Z 1×/0.25 FWD 56 mm objective and a Zeiss Axiocam 705 color camera. OFT area was measured using Fiji from maximum intensity projections.

### Zebrafish cardiac chamber size, cartilage, fin and eye distance measurements

For cardiac chamber size measurements, zebrafish larvae hearts were stopped in 0.2% tricaine and larvae were imaged on 3% methyl cellulose by Zeiss AxioZoom V16. Atrial and ventricular chamber dimensions were assessed by measuring two-dimensional areas from fluorescent images using Fiji [24], and values were normalized to control samples. Because this approach is based on a single imaging plane, it should be interpreted as an indirect estimate of chamber size rather than a direct quantification of chamber volume.

Alcian blue staining for facial and fin cartilage was performed according to previous protocols with minor alterations [26]. In brief, embryos were euthanized at 5 dpf before being fixed overnight at 4 °C in 4% PFA. Embryos were then washed with PBS-T and bleached with 3% H_2_O_2_ and 0.5% KOH for 30 min. Embryos were carefully washed in PBS-T three times before they were stained with 0.01% Alcian blue/60 mM MgCl_2_/70% ethanol (EtOH) overnight at 4 °C. Embryos were then incubated for 10 min at RT in 80% EtOH/10 mM MgCl_2_, 50% EtOH/10 mM MgCl_2_, and 25% EtOH/10 mM MgCl_2_, respectively. Further bleaching of the embryos was carried out with 3% H_2_O_2_ and 0.5% KOH for 15 min. Washes with 25% glycerol and 0.1% KOH were performed at least twice or until any remaining bubbles from the bleach disappeared. Samples were then stored at 4 °C in the dark in 50% glycerol and 0.1% KOH. For cartilage, fin and eye distance measurements, embryos were imaged ventrally on 3% methyl cellulose with a Zeiss AxioZoom V16 microscope. Distances and angles were measured using Fiji as previously described [53] and plotted.

### Zebrafish cardiac rate and contractility measurements

Zebrafish larvae were removed from the incubator and allowed to acclimate to room temperature for 10 min. Heart rates were then counted manually under Zeiss Stemi 508 microscope for 15 seconds and then multiplied by four to get beats per minute (bpm). For cardiac contractility measurements, zebrafish larvae were anesthetized in 0.005% tricaine for 3 min and mounted in 0.8% low melting point agarose (Invitrogen). Recordings were performed by Photometrics Prime BSI camera attached to an Olympus BX63 microscope at 100 fps. The ejection fraction and fractional shortening calculations were done upon ventricular diameter measurements by Fiji as previously described [27].

### Zebrafish trabeculation analysis

Zebrafish larvae at 6 dpf in the *Tg(myl7:GFP)* background were used. Samples were immunostained with an anti-GFP antibody (see S3 and S4 Tables) and imaged (interval 3 μm) using Zeiss LSM either 980 or 900 equipped with Plan-Apochromat 40×/1.4 objective. Cardiac trabeculation was quantified using a custom macro written in Fiji (ImageJ). Z-stack images of hearts were processed by conversion to 8-bit, followed by background subtraction, automatic thresholding, and binary mask generation. For each image, the ventricular lumen was manually delineated as a region of interest (ROI), after which particle analysis was performed to identify and quantify trabecular protrusions within the defined region with size filtering parameters applied to exclude noise and non-relevant structures. The custom Fiji macro is available upon request.

### Zebrafish transcriptome analysis and RT-qPCR

Sibling *tak1*^*+/−*^ and *tak1*^*+/+*^ adult zebrafish were used for breeding. The phenotypic *tak1*^*−/−*^ larvae were selected from the *tak1*^*+/−*^ breeding; while the *tak1*^*+/+*^ larvae were obtained from the *tak1*^*+/+*^ breeding. All experimental groups were stage-matched, and no evidence of developmental delay was observed. 3 dpf zebrafish larvae hearts were extracted as previously described [23]. Three groups of hearts (15 hearts each) were collected from *tak1*^*−/−*^ and *tak1*^*+/+*^ GFP-positive larvae. Total RNA (>200 nucleotides) was isolated using Qiagen Rneasy micro kit according to manufacturer’s protocol. Bulk RNA-seq was performed as a service by BGI, China. In brief, samples were paired end-sequenced using the DNBSEQ platform. After quality filtering, reads were aligned to reference sequence (GCF_000002035.6_GRCz11) using HISAT and Bowtie 2. Average mapping ratio with reference genome was 88.50%, average mapping ratio with genes was 74.56%. In total 23966 genes were identified. RNA-seq data was uploaded to Gene Expression Omnibus (GSE279246). Remaining tissue was collected from the larvae for verifying the genotype by Sanger sequencing. The sequencing chromatogram from phenotypic larvae revealed clear peaks with a single bp deletion as expected from *tak1*^*−/−*^ zebrafish. Differentially expressed genes were identified as genes with statistically significant difference in expression between *tak1*^*−/−*^ and *tak1*^*+/+*^ samples (*q*-value <0.05). Volcano plot was performed using VolcaNoseR (https://huygens.science.uva.nl/VolcaNoseR/). Human orthologues of zebrafish genes were selected using HGNC Comparison of Orthology Predictions (HCOP) (https://www.genenames.org/tools/hcop/). GO annotation analysis was performed using WebGestalt (https://www.webgestalt.org/) [28]. Gene enrichment was determined by calculating overlap between gene lists. Significant overlap was calculated using hypergeometric statistics (http://nemates.org/MA/progs/overlap_stats.html). A representation factor was calculated as the number of overlapping genes, divided by the expected number of overlapping genes drawn from two independent groups: RF = *x*/((n*D)/N), where *x* = number of overlapping genes, *n* = genes in group 1, *D* = genes in group 2, *N* = genes in genome (20,000). For RT-qPCR, total RNA from pools of ~15 zebrafish embryos was extracted using a combination of Trizol (Ambion) and a Direct-zol RNA miniprep kit (Zymo Research), and used to synthesize random-primed cDNA (SuperScript II Reverse Transcriptase, Invitrogen). Brilliant III Ultra-Fast SYBR Green QPCR Master Mix (Agilent Technologies) was used to amplify cDNA, and relative quantities were normalized to *actb1* expression. Samples were analyzed using a QuantStudio 5 real-time PCR system (Applied Biosystems). Primers are listed in S5 Table. For RT-qPCR, embryos at 3 dpf and 5 dpf for *tak1* and *tab2* mutants were used, respectively, as these were the stages when mutants were fully penetrant and phenotypically distinguishable from their heterozygous and WT sibling from the same breeding (heterozygous adult in-cross).

### RPE-1 cell culture, stimulation assay and transfection

RPE-1 (human telomerase reverse transcriptase-immortalized retinal pigment epithelial-1) cells (laboratory stock; originally derived from the immortalized hTERT-RPE1 cell line, ATCC, clone CRL-4000) were cultured in T75 flasks at 37 °C and 5% CO_2_ in DMEM (Gibco) supplemented with 10% fetal bovine serum (FBS, Gibco) and 1% penicillin-streptomycin (Sigma-Aldrich) and passaged with 1% Trypsin (Sigma-Aldrich) when the cells reached 80% confluency. RPE-1 cells were seeded as 2,000 cells/cm^2^ in DMEM (+/+) media in culture dishes. After 24 hours, cells were washed with 1× PBS and serum starved in serum-depleted DMEM for 48 hours. The cells were then stimulated with TGFB1 (R&D Systems, #240-B) or BMP2 (R&D Systems, #355-BM) ligand at a final concentration of 2 ng/mL and 100 ng/mL, respectively, as previously described [29]. The cells were then fixed with 4% PFA for IFM or lysed with SDS for western blot. The RPE-1 cells were transfected with plasmid constructs p3XFLAG-TAK1-WT, p3XFLAG-TAK1-P485L, p3XFLAG-TAK1-G168R which comprised WT TAK1 and two FMD patient mutations, respectively. Transfection was achieved using Lipofectamine 3000 Transfection Kit (Invitrogen, L3000-015, 2201452). Briefly, RPE-1 cells were seeded as 100,000 cells/well in 6-well plates with DMEM medium supplemented with 10% FBS and 1% penicillin-streptomycin for 24 hours. The cells were then serum starved for 48 hours followed by incubation with transfection mixture and 1.5 μg of respective plasmid DNA for 8 hours. Cells were then analyzed for IFM or western blot. For TAK1 inhibition assays, RPE-1 cells were seeded at 100,000 cells/well in 6-well plates with DMEM medium supplemented with 10% FBS and 1% penicillin-streptomycin for 24 hours. The cells were then serum starved for 48 hours and subsequently incubated with 500 nM 5ZO or the corresponding vehicle for 4 hours before being stimulated with TGFB1 or BMP2 as described above.

### P19CL6 mouse stem cell culture, cardiomyogenesis and TAK1 inhibition

CRISPR-Cas9 gene editing was used to generate mutants of P19CL6 stem cells (laboratory stock [30]; originally derived from mouse embryonal carcinoma tissue by Habara, Akemi and registered with Murofushi, Kimiko, Japan; ref nr. 24063467). For *Tak1*, an 85 bp deletion was introduced in *Tak1* that induced exon 2 skipping resulting in *Tak1* homozygous KO clone. For *Tab2*, a heterozygous *Tab2* KO clone was generated by inducing a 58 bp deletion on allele A leading to a premature stop codon at nucleotide 418. Additionally, a 10 and 25 bp deletion was introduced on allele B causing a premature stop codon at nucleotide 298. The P19CL6 WT and *Tak1* and *Tab2* mutants were cultured in T25 flask at 37 °C and 5% CO_2_ in MEM alpha medium (Gibco) supplemented with 10% FBS (Gibco) and 1% penicillin-streptomycin (Sigma-Aldrich) and passaged with 1% trypsin (Sigma-Aldrich) when reached at 80% confluency. For cardiomyocyte differentiation, the P19CL6 cells were seeded with a density of 1,000 cells/cm^2^ with an induction by 1% DMSO in the culture medium. Differentiation assay was performed as previously described [30]. For TAK1 inhibition assay, P19CL6 cells were seeded as 4,800 cells/mL in the presence of either 10 μM Takinib or 500 nM 5ZO or the vehicle and set to differentiate in the presence of 1% DMSO.

### Immunofluorescence microscopy analysis of RPE-1 and P19CL6 cells

RPE-1 and P19CL6 cells were grown on coverslips and washed with 1× PBS, fixed in 4% formaldehyde for 15 and 25 min, respectively, permeabilized in 0.2% Triton X-100 at room temperature for 12 min and blocked with 2% BSA for 30 min. Primary antibodies (S3 Table) were incubated overnight at 4 °C, and secondary antibodies (S4 Table) at room temperature for 45 min followed by 30 seconds incubation with DAPI. Images were visualized using an Olympus BX63 upright microscope with a DP72 digital camera. Olympus CellSens dimension software was used to measure the fluorescent intensities at the cilium-centrosome axis. The mean fluorescence values at the cilium-centrosome axis were set relative to the fluorescence values in background areas of the cytosol. Images were processed for publication using Adobe Photoshop CS6 (version 13.0) and data were presented using GraphPad Prism software (version 9). GATA4 and α-actinin quantification was performed by finding regions filled with nuclei via the DAPI channel, switching to the channel of interest and then quantifying the number of positive nuclei or the total fluorescent intensity of the frame, respectively.

### Seeding, differentiation and immunofluorescence microscopy analysis of gastruloids

Mouse embryonic stem cells (mESCs) required for gastruloid formation were procured from an 80% confluent T25 gelatin-coated flask. The old medium was aspirated followed by a wash in 10 mL Gibco pH7.4 PBS. Hereafter, the cells were trypsinized with TrypLE Express (Gibco, #12605010) and incubated for 5 min at 37 °C, 5% CO_2_. Trypsin was neutralized with the addition of 7 mL DMEM medium (Gibco, #11965092) + Trypsin Neutralization solution (Gibco, #R002100). The cells were then centrifuged at 170*g* at room temperature for 5 min. The pellet was washed twice by adding 10 mL PBS, then centrifuging the cells for 5 min at 170*g*. The cells were resuspended in 5 mL N2B27 medium. Using a kova-slide (Pierron, #13367.20), the cells were counted and seeded to 7,500 cells/mL, whereafter 40 μL was added to each well of a 96-well plate, making the final cell-count per well approximately 300. The cells were then incubated at 37 °C, 5% CO_2_ for two days. Following gastruloid seeding, 150 μL of fresh N2B27 medium + 3 μM CHIR99021 was added to the cells that were then incubated at 37 °C, 5% CO_2_ for one day. On day 3, 120 μL of the old medium was removed and replaced with further 150 μL of fresh N2B27 medium. At the beginning of day 4, gastruloid elongation was observed. 150 μL of the old medium was replaced with 150 μL of fresh N2B27+++ medium (30 ng/ml bFGF (R&D Systems, #233-FB), 5 ng/ml VEGF (Gibco, #PHC9394) and 0.5 mM Ascorbic Acid (Sigma, #255564) whereafter the 96-well plates were placed on an orbital shaker at continuous shaking at 100 rpm at 37 °C, 5% CO_2_. Fixed gastruloids were serially dehydrated in EtOH/PBS and embedded in paraffin. Lateral sections were collected for analysis. Sections were de-paraffinized with several washes in xylene, followed by serial gradient rehydration in EtOH. Sections were boiled in citrate buffer (pH 6.0) or Tris-EGTA (TEG) buffer (pH 8.0) for approximately 20 min and cooled. Blocking buffer (DAKO Real Antibody Diluent, #S2022) was added to the samples for 30 min before the sections were incubated with primary antibodies (S3 Table) diluted in blocking buffer overnight at 4 °C. Sections were then washed with 1× TBS (Tris-buffered saline) before incubating for 45 min in secondary antibodies (S4 Table) and DAPI diluted in blocking buffer. Washes with 1× TBS were performed again before a final wash in dH_2_O. Images were taken on an Olympus BX63 upright microscope as described for RPE-1 and P19CL6 cells.

### SDS-PAGE and western blotting analysis

RPE-1 and P19CL6 cells were washed in 1× PBS and lysed with 1% SDS (1% SDS, 1M Tris-HCl (pH 7.5)) and EBC (4 M NaCl, 1 M Tris-HCl (pH 7.5), 0.5 M EDTA, 0.5% NP-40) lysis buffers, respectively. Lysates were homogenized by sonication and centrifuged to collect the supernatant. Zebrafish larvae: approximately 15, 3 dpf larvae were dechorionated manually and yolk was removed in deyolking buffer (55 mM NaCl, 1.8 mM KCl, 1.25 mM NaHCO_3_) by pipetting and shaking using a thermomixer. Larvae were pelleted at 3,000 rpm for 1 min at 4 °C and washed twice with wash buffer (110 mM NaCl, 3.5 mM KCl, 2.7mM CaCl_2_, 10 mM Tris (pH 8.5)). Following the last wash, supernatant was removed and 80 μl of 1% SDS lysis buffer supplemented with protease inhibitor was added. Pellet pestle motor (VWR, #47747-370) was used to dissociate larvae. Lysates were stored in −80 °C until use. For all lysates, protein concentrations were determined using BioRad DC Protein Assay, OD was measured at 750 nm using a Beckmann Coulter DU spectrophotometer. SDS-PAGE and western blot was performed as previously described [31]. Blots were developed in FUSION-FX chemiluminescence system. Band intensities were analyzed by densitometric scanning using UN-SCAN-IT 6.1 software (Silk Scientific). Source data are available in S1 Raw Images. Please see S3 and S4 Tables for primary and secondary antibodies, respectively.

### Statistical analysis

Unless otherwise mentioned, statistical analysis was carried out using the Student *t* test (unpaired, two-tailed) for comparing the variation between two groups. All statistical calculations were performed on *n* = 3 or more. Significance levels were as follows: ^*^p < 0.05, ^**^p < 0.01, ^***^p < 0.001 and ^****^p < 0.0001, ns: not significant.

## Results

### Increased burden of rare *TAK1* and *TAB2* PAVs in syndromic CHD

**Fig 1:**
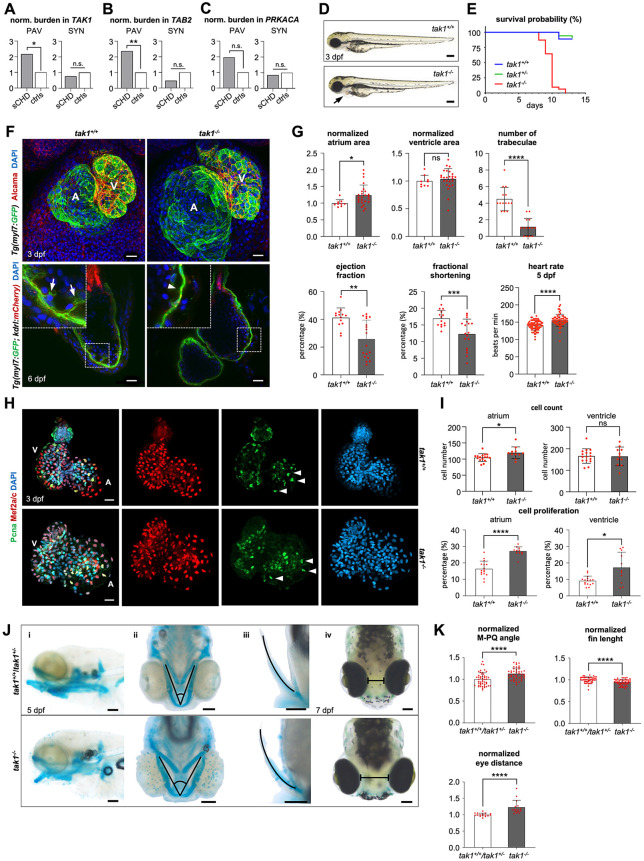
Phenotype of zebrafish *tak1* mutants. **(A–C)** Normalized burden of rare PAVs (left) and SYNs (right) in *TAK1* **(A)**, *TAB2* **(B)** and *PRKACA* **(C)**. The rare variants (MAF < 0.001) were identified by exome sequencing of 1,471 CHD patients with extracardiac anomalies (sCHD) and 45,082 controls (ctrls). Fisher’s exact test, *: *P* < 0.05, **: *P* < 0.01. **(D)** Gross morphology of 3 dpf *tak1*^*+/+*^ and *tak1*^*−/−*^ mutant zebrafish larvae highlighting pericardial edema (arrow). Scale bars, 0.2 mm. **(E)** Survival of *tak1*^*+/+*^ (*n* = 18)*, tak1*^*+/−*^ (*n* = 34) and *tak1*^*−/−*^ (*n* = 31) zebrafish larvae. **(F)** Upper panel: IFM of transgenic *Tg(myl7:GFP) tak1*^*+/+*^ and *tak1*^*−/−*^ zebrafish hearts with anti-GFP (green) and Alcama (red) antibodies at 3 dpf. A: atrium, V: ventricle. Lower panel: IFM of *Tg(myl7:GFP; kdrl:mCherry) tak1*^*+/+*^ and *tak1*^*−/−*^ zebrafish hearts with anti-GFP (green) and anti-mCherry (red) antibodies at 6 dpf. Ventricular trabeculae are indicated with arrows and trabeculation defects with an arrow head. Nuclei were stained with DAPI (blue). Scale bars, 20 μm. **(G)** Quantification of normalized atrium area, ventricle area (*n* = 9, 25), ejection fraction (%) and fractional shortening (%) (*n* = 14, 18) at 3 dpf, heart rate (*n* = 72, 62) at 5 dpf and number of trabecular ridges (*n* = 14, 14) at 6 dpf. Statistical tests: *t* test. **(H)** IFM of cardiomyocy*t*e nuclei and proliferating cells, using anti-Mef2a/c (red) and anti-Pcna (green) antibodies, respectively in 3 dpf *tak1*^*+/+*^ (upper) and *tak1*^*−/−*^ (lower) zebrafish heart extracts (DAPI, blue). Scale bars, 20 μm. Arrow heads show proliferating cells. **(I)** Quantification of number of cardiomyocytes (Mef2a/c-positive cells) and cell proliferation index in atrium and ventricle of 3 dpf *tak1*^*+/+*^ and *tak1*^*−/−*^ zebrafish hearts (*n* = 16, 11). **(J)** Extracardiac defects in *tak1* mutants. Bright-field images of *tak1*^*+/+*^*/tak1*^*+/−*^ (upper panel) and *tak1*^*−/−*^ (lower panel) larvae, showing **(i)** lateral views of cartilage stained with Alcian blue at 5 dpf. **(ii)** Meckel’s-palatoquadrate (M-PQ) angle measurements of cartilage. Black lines show the measured angle in larvae. **(iii)** Measurement of pectoral fin length. Black line shows measured fin length. **(iv)** Eye distance measurement at 7 dpf. Black line shows the measured eye distance in larvae. Scale bars, 0.1 mm. **(K)** Quantification of measurements obtained from panels ii (*n* = 49, 44), iii (*n* = 47, 46), iv (*n* = 16, 15) in **(j)**. *t* test, *: *P* < 0.05, **: *P* < 0.01, ***: *P* < 0.001, ****: *P* < 0.0001, ns: no*t* significant. Source data are available in S1 Data.

To confirm the association between *TAK1*, *TAB2, PRKACA* and heart defects in a pediatric cohort, we initially analyzed exome sequencing (ES) data from a cohort of 1,471 sCHD patients, 2,405 nsCHD patients and 45,082 controls [19]. We identified 52 rare (MAF < 0.001) variants in cases and 483 rare variants in controls (S1 Table). CADD scores of PAVs ranged from 9.2 to 40 (mean = 25.3) in cases and from 0.8 to 38 (mean = 23.4) in controls (S1 Table; S1A and S1B Fig). We calculated the burden of rare SYNs and PAVs between the two patient cohorts and controls, and analyzed the difference using Fisher’s exact test. In ES data obtained from sCHD patients, we observed an approximately 2-fold increase in the burden of rare PAVs for *TAK1, TAB2* and *PRKACA*, when compared to controls. This difference was statistically significant for *TAK1* and *TAB2* (*P*-value 0.042 and 0.0081, respectively) (Fig 1A–1C). In ES data obtained from nsCHD, we observed no difference in variant burden for any of the three genes. As a negative control, we compared the burden of rare SYNs and did not observe any significant difference between nsCHD and controls (Fig 1A–1C). Focusing the sCHD burden analysis on potentially disease-causing variants (defined as variants with CADD score > 25) increased the burden to 3.3 and 11.5 for *TAK1* and *TAB2*, respectively (with P-values of 0.022 and <0.0001) (S1C Fig). For *PRKACA* we did not identify variants with CADD > 25 in sCHD cases. Together, these results confirm that rare pathogenic variants in *TAK1* and *TAB2* are associated with sCHD.

### Homozygous mutation of *tak1* and *tab2* is lethal and causes heart defects

In mouse models, homozygous deletion of *Tak1* and *Tab2* is embryonic lethal at E10.5 and E12.5, respectively [32,33]. To analyze the mechanisms of TAK1 in in vivo heart development, we generated zebrafish lines with 1 and 8 bp deletions in the coding sequence of *tak1* and *tab2*, respectively, using the CRISPR-Cas9 technique. Both variants lead to a shift in the reading frame of the gene, resulting in a premature stop codon (S1D–S1G Fig).

Zebrafish *tak1*^*−/−*^ homozygote mutants (will be referred to as *tak1* mutants hereafter) showed normal cardiac progenitor patterning at 14 hpf (S2A Fig). *Tak1* mutants began to display cardiac defects in a subset of embryos at 2 dpf, followed by the appearance of pericardial edema at 3 dpf with full penetrance. The presence of pericardial edema enabled reliable identification of homozygous mutants from progeny of heterozygous crosses for subsequent analyses. Accordingly, most experiments were conducted at 3 dpf or at later stages for structures that emerge subsequently. Heterozygote siblings were phenotypically inseparable from the WT siblings (Fig 1D). Survival of *tak1* mutants was severely compromised compared to WTs and heterozygotes, with no *tak1* mutant surviving past 12 dpf (Fig 1E). Immunofluorescence microscopy (IFM) analysis of the cardiomyocytes revealed defects in cardiac morphology. We observed large variability in ventricle and atrium size in *tak1* mutants, with the atrium being significantly enlarged at 3 dpf (Fig 1F and 1G). When we assessed the hearts at 6 dpf, we observed a significantly reduced number of trabecular ridges in *tak1* mutants (Fig 1F and 1G). The *tak1* mutants had defects in heart function, as measured by reduced fractional shortening and ejection fraction at 3 dpf, and in addition, we observed increased heart rate in *tak1* mutants at 5 dpf (Fig 1G).

To investigate the changes in cardiac chamber sizes in *tak1* mutants, we quantified atrial and ventricular cardiomyocyte numbers, measured cell sizes, and compared them to those of *tak1*^*+/+*^ larvae. We observed significantly more cells in the atrium of *tak1* mutants (Fig 1H and 1I), but did not observe any difference in cell size (S2C and S2D Fig). Consistently, the atrial cardiomyocyte proliferation index was significantly higher in *tak1* mutants at 3 dpf (Fig 1H and 1I). Although ventricular cardiomyocyte proliferation was also increased, this did not translate into a higher ventricular cell number (Fig 1I). This discrepancy can be due to survival, or differentiation of proliferating cells in the ventricle. Additionally, the high variability observed in ventricular proliferation highlights the heterogeneity of proliferation outcomes in this chamber of *tak1* mutants.

To assess gross effects on myofibrils, we measured the distance between sarcomeric z-discs in atrium and ventricle but did not identify any difference between *tak1* mutants and *tak1*^*+/+*^ larvae (S2E and S2F Fig). Cardiac valve defects are hallmarks of THS and are also found in a number of patients with *TAK1* mutations [11,13]. Thus, we assessed the atrioventricular (AV) valves in *tak1* (and *tab2*, see below) mutants, but did not observe significant difference in number of cells in the valves (S2G and S2H Fig). Finally, we tested the *tak1* mutants for apoptotic changes by acridine orange staining and analysis of expression of *bcl2* and *baxa*, but there was no difference in apoptosis markers between *tak1* mutant and *tak1*^*+/+*^ larvae (S2I and S2J Fig).

To further analyze the effect of defective Tak1 signaling in heart development, we created *tab2*^*−/−*^ homozygous mutants (will be referred to as *tab2* mutants hereafter). These mutants phenocopied *tak1* mutants with a slight delay. *Tab2* mutants began to display heart defects and pericardial edema in a subset of embryos at 2 dpf and 3 dpf, respectively, which reached full penetrance by 5 dpf. Thus, most experiments were conducted at 5 dpf. We noted that *tab2* mutants, similar to *tak1* mutants at 5 dpf, display a protruding mouth (S2B and S3A Figs). We observed less pronounced lethality in *tab2* mutants, beginning from 7 dpf, with survival probability of 40% at 19 dpf (S3B Fig). However, *tab2* mutants did not survive to adulthood, suggesting that significant mortality of mutants occurs between 20 dpf and adulthood. Similar to *tak1* mutants, cardiac morphological defects in chamber size, looping and trabeculation were also observed in the *tab2* mutants; which displayed enlarged atria at 5 dpf and severely reduced ventricular trabeculation observed at 6 dpf (S3C and S3D Fig). We further observed a significant increase in cell number within the atrium of *tab2* mutants (S3E and S3F Fig), accompanied by a markedly elevated cardiomyocyte proliferation index in this chamber at 5 dpf (S3G Fig). Cardiac function was also compromised in *tab2* mutants, marked by increased heart rate and reduced contractility at 5 dpf (S3D Fig). Cardiac valve defects are more pronounced in patients with *TAB2* mutations, occurring in over 75% of cases, compared to those with *TAK1* mutations [11]. Consistently, *tab2* zebrafish mutants displayed AV valve developmental defects, marked with a significant decrease in cell number (S3H and S3I Fig).

The superfamily of TGFB signaling regulates outflow tract (OFT) development [34–36]. To determine whether OFT development is affected in *tak1* and *tab2* mutants, we performed in situ hybridization marking *elastin b (elnb)* and observed a hypoplastic OFT in these zebrafish, indicating a role for Tak1/Tab2-dependent signaling in OFT development (S4A and S4B Fig).

Finally, to validate the cardiac phenotypes in *tak1* mutant larvae, WT and *Tg(myl7:GFP)* zebrafish embryos were treated with two pharmacological inhibitors of Tak1, Takinib and 5ZO. Both treatments reproduce key *tak1* mutant phenotypes, including pericardial edema and atrial enlargement (S5A and S5B Fig). Compared to Takinib treatment, 5ZO-treated zebrafish displayed a more uniform and consistent phenotype (observed in 100% of larvae) that very closely recapitulated that of *tak1* mutants. We therefore used 5ZO to assess trabeculation and found that 5ZO-treated zebrafish exhibited a reduced number of trabecular ridges, consistent with *tak1* mutants (S5C Fig). Collectively, these findings strengthen the causal link between Tak1 function and the observed cardiac defects. We note, however, that some aspects of the mutant phenotype may arise as secondary consequences of earlier defects, and future studies are needed to further define the precise onset and primary versus secondary nature of these defects.

### *tak1* and *tab2* mutants exhibit abnormalities in both craniofacial structure and fin development

Facial dysmorphisms and limb defects are among the clinical characteristics of FMD, CSCFS and THS [11,13,14,37]. To test if *tak1* and *tab2* mutants phenocopy the human syndromes, we compared craniofacial features and length of the pectoral fins in homozygous mutants and WT/heterozygous siblings. We measured the Meckel’s‑palatoquadrate (M‑PQ) angle and eye distance, which reflect the relative positioning and patterning of craniofacial structures and allow detection of subtle defects in jaw and facial morphogenesis. Homozygous *tak1* and *tab2* mutants both showed significant increase in Meckel’s-palatoquadrate angle at 5 dpf, while eye defects were observed in both mutants at 7 dpf (Figs 1J, 1K, S3J, and S3K). Altogether, these data suggest craniofacial abnormalities in the mutants. At 5 dpf, the pectoral fin length, but not other fin measurements, was significantly reduced in both *tak1* and *tab2* mutants, consistent with features of brachydactyly. We did not observe significant phenotypes in adult heterozygous *tak1*^*+/−*^ and *tab2*^*+/−*^ zebrafish and the lethality of homozygous genotypes prevented us from analyzing adult *tak1* and *tab2* mutants. Together with the observed cardiac defects, these results suggest that the *tak1* and *tab2* mutants phenocopy several of the clinical characteristics of human FMD, CSCFS and THS.

### *tak1* mutants display downregulation of genes encoding core cardiac transcription factors, sarcomeric proteins and extracellular matrix proteins

**Fig 2:**
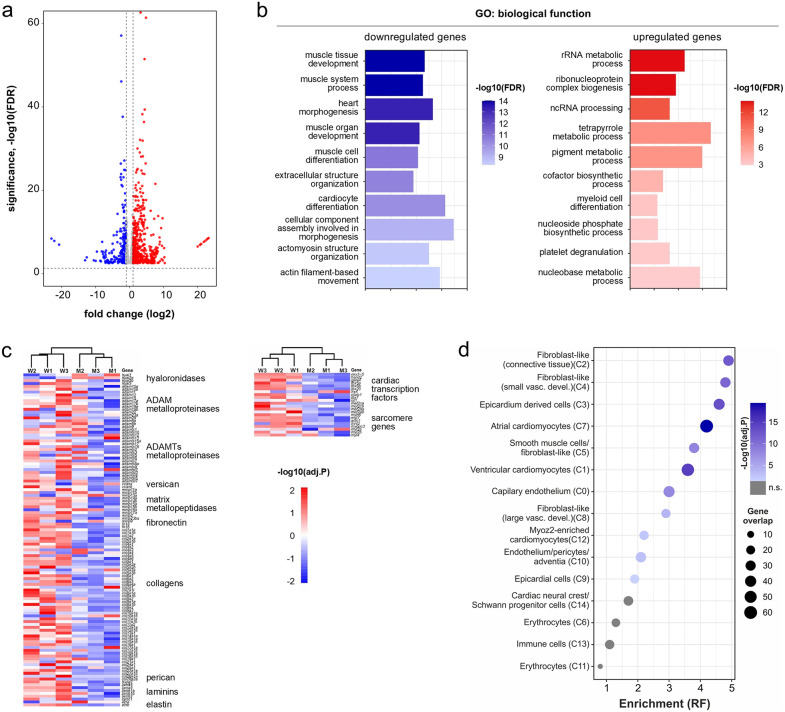
Transcriptome analysis of dissected zebrafish hearts. RNA sequencing was performed on hearts dissected from 3 dpf *tak1*^*+/+*^ and *tak1*^*−/−*^ zebrafish larvae. **(A)** Volcano plot of 581 down (blue) and 863 upregulated (red) genes. **(B)** Gene ontology (GO) enrichment analysis of 474 down (left) and 712 upregulated (right) genes (human orthologues to zebrafish genes). Enrichment was tested using WebGestalt [28,72]. **(C)** Heatmap showing the expression values of selected genes associated with ECM (left) and cardiomyogenesis (right). **(D)** Enrichment of downregulated genes in the expression signature of 15 cell-types in 6.5–7 WPC human developing hearts [40]. Enrichment is shown as representation factor (RF). A hypergeometric distribution was used to test the significance. P-values were adjusted using Bonferroni correction. Source data are available in S1 Data.

To identify developmental mechanisms regulated by Tak1, we compared the cardiac transcriptome of WT and mutant *tak1* larvae. To this end, we dissected the hearts of *tak1*^*+/+*^ and *tak1*^*−/−*^ larvae with *Tg(myl7:GFP)* background at 3 dpf and analyzed the transcriptome of the hearts by RNA sequencing. We identified 581 downregulated and 863 upregulated genes (Fig 2A). These genes correspond to 474 and 712 human orthologues, respectively (listed in S2 Table). Within the list of downregulated genes, we observed enrichment of genes known to cause CHD in human patients and mouse models [38,39], suggesting that TAK1 promotes the expression of several genes known to cause CHD (S6A Fig). We observed no significant enrichment of known CHD genes within the list of upregulated genes. Gene-ontology (GO) analysis of the human orthologues to downregulated genes, showed enrichment of genes involved in heart morphogenesis, cardiomyogenesis, muscle tissue development and ECM (Fig 2B, left panel). Significantly downregulated genes include genes encoding cardiac transcription factors and sarcomeric proteins as well as genes involved in structure and metabolism of the ECM (Fig 2C). Downregulation of the cardiac transcription factor genes *tbx5b*, *gata4* and *hand2*, sarcomeric genes *actc1a* and *myl7*, and the ECM-related gene *has2* in *tak1* mutants was confirmed by RT-qPCR analysis of dissected hearts from 3 dpf larvae (S6B Fig). Consistently, comparison of the cardiac expression level of the same set of genes between *tab2*^*+/+*^ and *tab2* mutant larvae at 5 dpf gave similar results (S6C Fig), supporting a functional interaction between *tak1* and *tab2* in heart development. GO analysis of upregulated genes suggested that these genes were involved in basic cellular processes, differentiation of myeloid cells and platelet degranulation (Fig 2B, right panel). Thus, a significant part of the upregulated genes may be false positives, caused by entrapment of more blood cells in the dilated atrium of mutant hearts, compared to WT hearts.

Lastly, to identify possible cardiac cell types affected by downregulated genes, we calculated the enrichment of downregulated genes in cell-specific gene-signatures obtained from single cell sequencing of human embryonic hearts [40]. We observed significant enrichment of downregulated genes in most cell types of the developing heart, except neural crest cells (Fig 2D). Most pronounced enrichment was observed in cardiac fibroblasts, epicardium-derived cells, cardiomyocytes and smooth muscle cells. Importantly, these annotations reflect transcriptional similarity to mammalian embryonic cardiac cell states rather than direct identification of zebrafish cell types, and the observed fibroblast-associated signal may therefore correspond to epicardial or bulbus arteriosus-derived cell populations in the zebrafish heart, as zebrafish hearts at this stage are not considered to contain a distinct fibroblast population. For upregulated genes, we only observed enrichment in gene-sets specific for erythrocytes and immune cells, supporting that a significant fraction of upregulated genes is related to blood cells (S6D Fig).

Based on our cardiac transcriptome analysis, TAK1 appears to influence a subset of gene networks associated with myocardial development, cardiac ECM, and cardiac smooth muscle cells, although these changes likely reflect both direct TAK1-dependent effects and secondary responses to altered cardiac function.

### TAK1 localizes to primary cilia and is activated at this site by TGFB/BMP stimulation and during cardiac development

**Fig 3:**
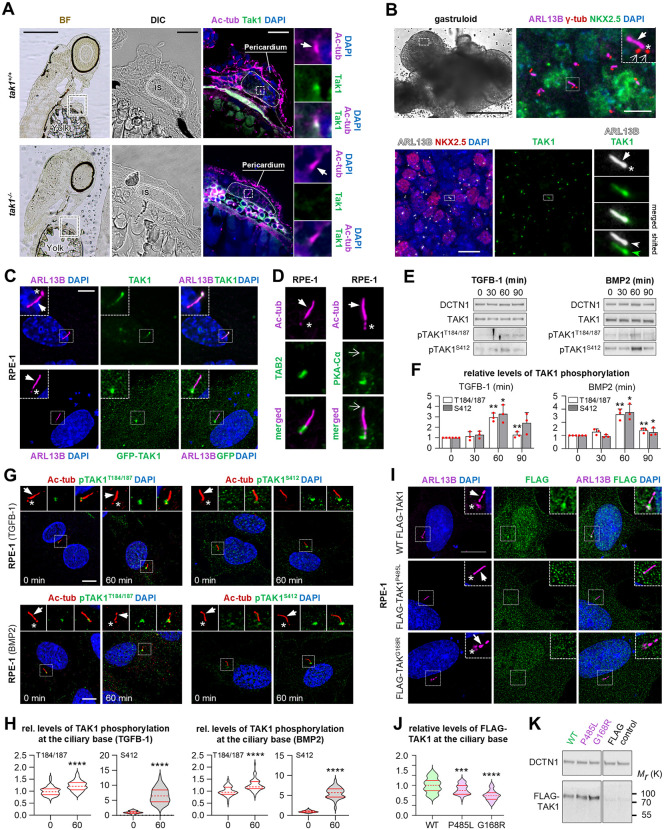
TAK1 localizes to the primary cilium and is activated at this site by TGFB/BMP stimulation. **(A)** Bright field DIC and IFM of heart sections from 3 dpf *tak1*^*+/+*^ (top) and *tak1*^*−/−*^ (bottom) zebrafish larvae stained with Tak1 (green) and acetylated tubulin antibody (Ac-tub, magenta). Dotted box in BF images represent the area interrogated via DIC and IFM. Arrow points to the cilium. IS: Intracardial space. Brightfield image scale bar, 200 μm, DIC and IFM scale bars, 20 μm. **(B)** Upper left panel: DIC microscopy image of day 7 gastruloid, white box indicates ROI. Scale bar, 0.5 mm. Upper right panel: presence of primary cilia (ARL13B, magenta) in cardiac progenitor cells (NKX2.5, green). Centrioles were stained with γ-tubulin (red). Large image scale bars, 10 μm, small image scale bars, 2.5 μm. Lower row panels: NKX2.5 (green) positive cardiac progenitor cells and ARL13B (white) stained primary cilia. TAK1 (green) is observed at primary cilia of day 7 gastruloids shown with both merged and shifted images. Colored arrows in shifted image denotes the different channels. Large image scale bars, 10 μm. **(C)** IFM of endogenous TAK1 (green) and GFP-tagged TAK1 (green) at the primary cilium (ARL13B, magenta) in RPE-1 cells. Large image scale bar, 5 μm, small image scale bar, 2.5 μm. **(D)** IFM images of TAB2 and PKA-Cα, (green) at the primary cilium (Ac-tub, magenta). Scale bar, 2.5 μm. **(E)** Representative images of western blot analysis of TAK1, pTAK1^T184/187^ and pTAK1^S412^ at indicated time points in RPE-1 cells upon TGFB-1 (left) and BMP2 (right) stimulation. DCTN1 was used as a loading control. **(F)** Quantification of relative levels of TAK1 phosphorylation with TGFB-1 (left) and BMP2 (right) ligand stimulation from **(E)** (*n* = 3). One-way ANOVA, *: *P* < 0.05, **: *P* < 0.01. **(G)** Representative IFM images of pTAK1^T184/187^ and pTAK1^S412^ (green) at time point 0 and 60 min upon TGFB-1 (upper panels) and BMP2 (lower panels) stimulation in RPE-1 cells. Cilium is marked with Ac-tub (magenta). Scale bars, 10 μm. **(H)** Quantification of relative levels of fluorescent intensities of pTAK1 proteins at the ciliary base upon ligand stimulation from **(G)** (*n* = 3, 20 cilia per replicate). *t* tes*t*. **(I)** Representative IFM images of FLAG-tagged WT TAK1 and TAK1 FMD patient variants (green) co-stained with ARL13B (magenta) in transfected RPE-1 cells. Large image scale bar, 10 μm, small image scale bar, 2.5 μm. **(J)** Quantification of relative levels of FLAG-tagged WT TAK1 and mutant TAK1 at the ciliary base from **(I)**. *t* tes*t*, ***: *P* < 0.001, ****: *P* < 0.0001. **(K)** Western blot analysis showing expression levels of FLAG-tagged WT TAK1 and TAK1 FMD patient variants in transfected RPE-1 cells. In IFM images, closed arrows and asterisks indicate ciliary axoneme and base, respectively. Open arrows point to the centrioles and nuclei were stained with DAPI (blue). Source data are available in S1 Data.

In the developing heart, canonical TGFB/BMP signaling is coordinated by the primary cilium [41,42]. To investigate whether TAK1 similarly functions at the primary cilium during cardiogenesis, we initially analyzed the subcellular localization of Tak1 in zebrafish at 3 dpf. Zebrafish tissue sections were analyzed via IFM showing that Tak1 predominantly concentrates to the basal region of primary cilia in cardiac tissue (Fig 3A, top row), whereas immunoreactivity is absent in hearts dissected from *tak1* mutants (Fig 3A, bottom row), validating antibody specificity and ciliary Tak1 localization. Tak1 was also present at primary cilia in extracardiac tissues (S7A Fig), indicating that ciliary Tak1 functions beyond the heart and offering a mechanistic link to the extracardiac phenotypes associated with TAK1 variants in FMD, CSCFS and THS.

To further substantiate ciliary localization of TAK1 during cardiomyogenesis, we performed IFM analyses in day 7 gastruloids (Fig 3B, upper left panel), which differentiate into several progenitor cell populations, including heart field cells, in a 3D manner (S7B Fig) [43,44]. Our investigation revealed discrete regions within the gastruloids enriched in the cardiac transcription factor, NKX2–5, and the first heart field marker HCN4 [45] (S7C Fig), indicating the presence of cardiac progenitors [45,46]. Further analysis revealed that NKX2–5-positive regions developed well-defined Troponin-I-positive cardiomyofibrils (S7D Fig). In addition, both clusters of cardiac progenitor cells (Fig 3B, upper right panel) and cells located outside these clusters (S7E Fig) formed primary cilia, which serve as the principal site for TAK1 localization (Figs 3B, lower row and S7E). These discoveries align with our observations in the developing heart of zebrafish larvae, pinpointing the function of ciliary TAK1 in development of both the heart and extracardiac tissue. Furthermore, these novel findings underscore the utility of gastruloids as a system through which the involvement of primary cilia in early mammalian development can be delineated.

To support these findings and examine ciliary TAK1 activity in the context of cardiomyogenic stimuli, we proceeded to investigate TGFB/BMP-mediated TAK1 activation in RPE-1 cells, which are well-established model cells for developmental signaling and primary cilia studies [6]. We initially conducted IFM analysis, uncovering significant accumulation of both endogenous and GFP-tagged TAK1 at the ciliary base region, and in some cases with notable distribution along the primary cilium (Fig 3C). These observations were validated using markers for the ciliary base, basal-body distal appendages and IFT88, which marks the ciliary base as well as the length of the cilium (S8A–S8C Fig) Furthermore, we observed prominent ciliary base localization of the essential upstream regulators of TAK1 activity, TAB2 and PKA-Cα, which also localized to the ciliary tip (Fig 3D). Localization of PKA-Cα to primary cilia aligns with previous findings on the role of this subunit in the regulation of Hedgehog (Hh) signaling in the cilium, where it phosphorylates and inactivates GLI2/3 transcription factors to repress expression of Hh target genes [7,47]. Furthermore, TAK1 and TAB2 were recently detected in cilia proteomic studies [48,49], but their ciliary localization was not validated, and their function was not examined in these studies.

Next, western blot and IFM analyses were carried out to evaluate TGFB/BMP-mediated phosphorylation of TAK1 at T184/187 and S412, which marks its activation mediated by TAB2 and PKA-Cα, respectively [10,50]. To this end, RPE-1 cells were subjected to stimulation over a time interval of 90 min with TGFB-1 and BMP2, which play crucial roles in cardiac development, including intricate processes of cardiomyogenesis [41]. Both ligands elicited phosphorylation of TAK1 at T184/187 and S412, reaching peak levels at 60 min of stimulation (Fig 3E and 3F), and at this point, increased phosphorylation levels were observed specifically at the base of the primary cilium (Fig 3G and 3H). Because TGFB/BMP receptors function at the level of primary cilia [6,41], these results support the conclusion that cardiomyogenic signaling molecules of the TGFB/BMP family operate through cilia-localized TAB2 and PKA-Cα to activate TAK1 at this site.

As individuals with TAK1 mutations may exhibit the multi-system syndrome FMD [14], we investigated the potential impact of patient-specific mutant forms of TAK1 on its localization to primary cilia by transiently transfecting RPE-1 cells with plasmids coding for WT FLAG-TAK1 or plasmids coding for patient-specific FLAG-TAK1 variants G168R and P485L [14]. IFM analysis of transfected cells validated the ciliary localization of WT TAK1, whereas the patient-specific variants showed impaired recruitment to this site (Fig 3I–3K). Thus, TAK1 mutations identified in FMD patients appear to influence the localization of TAK1 to the primary cilium, potentially compromising cardiogenesis through impaired ciliary signaling.

### TAK1 is required for in vitro cardiomyocyte differentiation

**Fig 4:**
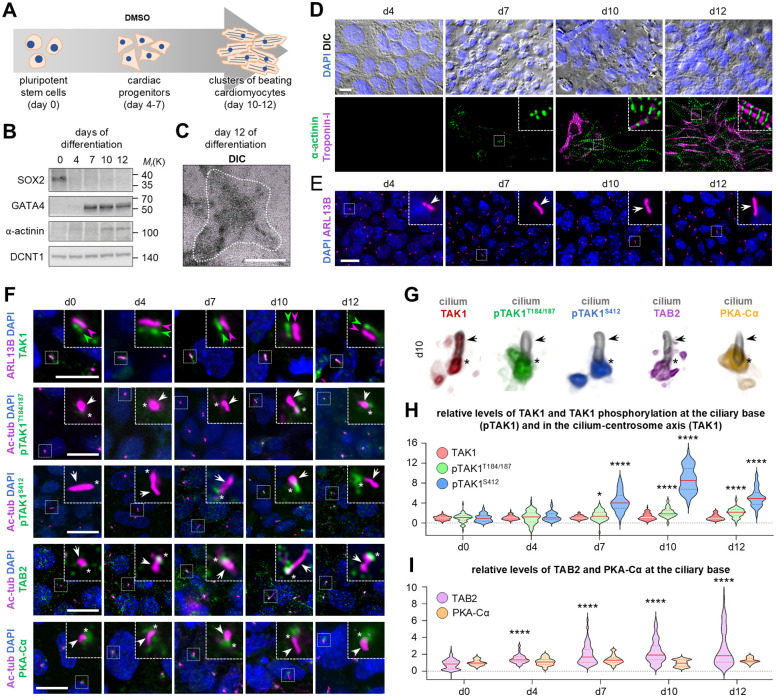
TAK1 is activated at the primary cilium during in vitro cardiomyocyte differentiation. **(A)** Graphical illustration of cardiomyocyte differentiation from day 0 to day 12 upon stimulation of pluripotent stem cells (P19CL6) with 1% DMSO. **(B)** Representative western blot analysis of SOX2, GATA4 and α-actinin protein levels in P19CL6 cells upon DMSO stimulation from day 0 to day 12. DCTN1 was used as a loading control. **(C)** DIC of a beating cluster of cardiomyocytes (within dotted white lines) at day 12 of DMSO-stimulated P19CL6 cells. Scale bar, 1 mm. **(D)** DIC and IFM images showing P19CL6 cells undergoing differentiation from day 4 to day 12. Upper panels: differential interference contrast (DIC) microscopy with staining of nuclei (DAPI, blue). Lower panels: IFM images showing ⍺-actinin (green) and Troponin-I (magenta) in the same area as the upper panels. Large image scale bar, 10 µm, small image scale bar: 2.5 μm. **(E)** Representative IFM images displaying primary cilia (arrows, ARL13B, magenta) during cardiomyocyte differentiation from day 4 to day 12. Large image scale bar, 10 μm, small image scale bar: 1 μm. (**F)** Representative IFM images showing expression of TAK1 (shifted), pTAK1^T184/187^, pTAK1^S412^ and upstream components TAB2 and PKA-Cα (all in green), co-stained with ciliary markers (ARL13B or Ac-tub, magenta) in P19CL6 cells. Arrow marks the cilium and asterisks denotes the ciliary base region. Scale bar, 20 μm. **(G)** Voxel view of primary cilia (gray) along with TAK1, pTAK1^T184/187^, pTAK1^S412^, TAB2 and PKA-Cα, colored as red, green, blue, magenta, and yellow, respectively, at day 10 of P19CL6 cell differentiation. **(H, I)** Quantification of relative levels of TAK1 (*n* = 4, 60 cilia) and activated TAK1 proteins (*n* = 3, S412: 65 cilia, T184,187: 60 cilia) (H) and TAB2, PKA-Cα (I) in the cilium-centrosome axis and at the ciliary base from **(F)**. Statistical analysis was performed via Kruskal–Wallis test for TAK1 proteins and PKA-Cα, one-way ANOVA was performed for TAB2, *: *P* < 0.05, ****: *P* < 0.0001. Source data are available in S1 Data.

To corroborate our findings in zebrafish, gastruloids, and RPE-1 cells, and to further investigate the role of TAK1 in cardiomyocyte differentiation, we selected P19CL6 teratocarcinoma-derived pluripotent stem cells. These cells provide a well-established model for studying early cardiomyogenesis [30,51–53], and have previously been used to link TAK1 with BMP-mediated cardiomyocyte differentiation [18]. Upon DMSO treatment, P19CL6 cells exit pluripotency within a few days, initiating cardiomyogenesis, as indicated by reduced SOX2 expression and increased GATA4 expression, a key transcription factor driving cardiac development (Fig 4A and 4B). By day 12, the cells form functional clusters of beating cardiomyocytes with distinct striated sarcomeric patterns of Troponin I and ⍺-actinin (Fig 4A–4D). Throughout differentiation, the cells also display primary cilia (Fig 4E), which are essential for cardiomyogenesis in this stem cell model [30,41].

During P19CL6 cardiomyogenesis, we observed a substantial enrichment of TAB2 as well as phosphorylation of TAK1 at T184/187 and S412 at the primary cilium, reaching peak levels around day 10 of differentiation (Fig 4F–4I). In contrast, the levels of total TAK1 and PKA-Cα at the cilium remained relatively stable throughout the differentiation process (Fig 4F–4I). Highlighting day 10 of differentiation, 3D imaging further delineated localization of TAK1 around the ciliary base as well as along the entire length of the cilium in a punctate pattern, whereas PKA-Cα, TAB2 and phosphorylated versions of TAK1 predominantly localized to the ciliary base region (Fig 4G). Together, these findings suggest that TAB2 is recruited to the cilium during differentiation, where, in conjunction with PKA-Cα, TAK1 phosphorylation and activation is mediated in the context of cardiomyogenesis.

**Fig 5:**
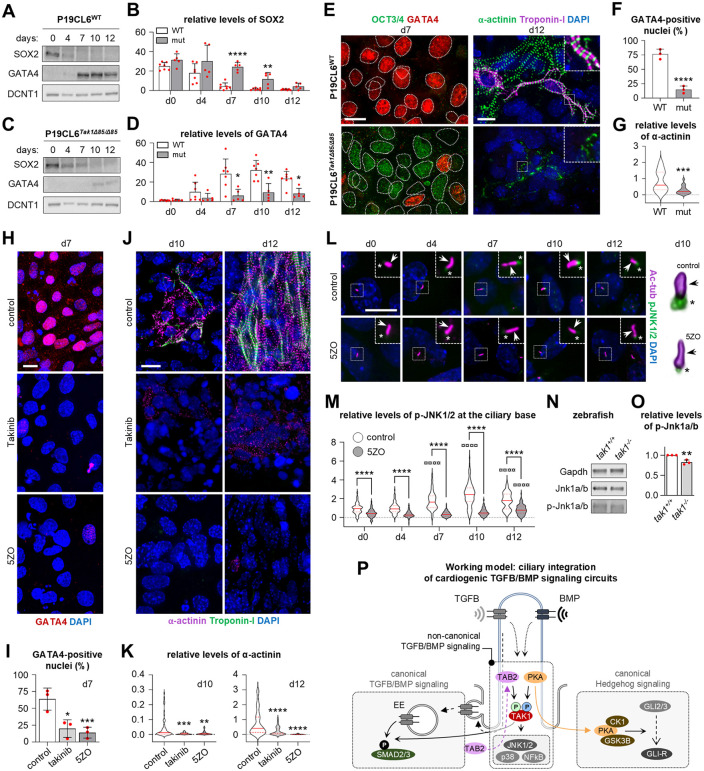
Mutation of TAK1 or inhibition of TAK1 activity impairs in vitro cardiomyocyte differentiation. **(A, C)** Western blot analysis at day 0, 4, 7, 10 and 12 of differentiation using antibodies against SOX2 and GATA4 from DMSO-treated P19CL6^WT^ and P19CL6^*Tak1Δ85/Δ85*^ cells, respectively. DCTN1 was used as a loading control. **(B, D)** Quantification of relative levels of SOX2 and GATA4 protein from **(A and C)**, One-way ANOVA. **(E)** Representative IFM images of OCT3/4 (green) and GATA4 (red) localization at day 7 (left panels) and α-actinin, Troponin-I at day 12 (right panels) in DMSO-induced P19CL6^WT^, P19CL6 Tak1^KO^ cells. Scale bars, 10 µm. **(F)** Percentage of GATA4 positive nuclei from E (left panel) (*n* = 3, P19CL6^WT^: 1,005 nuclei, P19CL6^*Tak1Δ85/Δ85*^: 872 nuclei). *t* test. **(G)** Quantification of relative levels of α-actinin from day 12 from **(E)** (right panel) (*n* = 3, 18 areas). *t* test. **(H, J)** Representative IFM images of GATA4 (red) at day 7 of differentiation (H) and α-actinin (green) and Troponin-I (magenta) at day 10 and 12 of differentiation (J) from P19CL6^WT^ cells treated without (control) and with TAK1 inhibitors (Takinib and 5ZO). Scale bars, 10 µm. **(I, K)** Percentage of GATA4 positive nuclei from **(H)** (*n* = 3, Vehicle: 2,980 nuclei, 5ZO: 3,356 nuclei, Takinib: 2,741 nuclei) and relative levels of α-actinin from **(J)** (*n* = 3, D0: 60 areas, D12: 90 areas), *t* test. **(L)** Representative IFM images of pJNK1/2 (green) at the primary cilium (magenta) from control and 5ZO-treated DMSO-induced P19CL6^WT^ cells from day 0 to day 12. Scale bar, 10 μm. Right panel: Representative voxel view of pJNK1/2 at the primary cilium at day 10 of P19CL6 differentiated cells in control and 5ZO-treated cells. **(M)** Relative levels of pJNK1/2 at the ciliary base from **(L)** (*n* = 3, 90 cilia), statistical analysis was performed via one-way ANOVA and two-tailed Student *t* test. **(N)** Western blot analysis of 3dpf tak1^+/+^ and tak1^−/−^ whole-embryo lysates using antibodies against p-Jnk1 and Jnk1. GAPDH was used as a loading control. **(O)** Quantification of relative levels of p-Jnk1a/b from **N. (P)** Working model for TAB2 and PKA-mediated activation of TAK1 and its downstream signaling components at the primary cilium to induce cardiogenesis. *: *P* < 0.05, **: *P* < 0.01, ***: *P* < 0.001, ****: *P* < 0.0001. ¤¤¤¤: *P* < 0.0001 compared to the respective means at day 0 (one-way ANOVA). Source data are available in S1 Data.

To examine the role of TAK1 in cardiomyogenesis in more detail, we next generated CRISPR-Cas9 P19CL6 mutants of *Tak1* containing a deletion, P19CL6^*Tak1Δ85/Δ85*^, resulting in skipping of exon 2, thus causing in-frame deletion of amino-acid residue 41–77 within the kinase domain of TAK1 (S9A and S9C Fig). The mutant cells exhibited prolonged expression of SOX2 and a delayed onset of GATA4 expression compared to WT clone cells (P19CL6^WT^) during the DMSO stimulation period (Fig 5A–5D). At day 7, while the majority P19CL6^WT^ cells were GATA4 positive, most P19CL6^*Tak1Δ85/Δ85*^ remained OCT3/4-positive and GATA4-negative (Fig 5E and 5F). Additionally, *Tak1* mutants displayed significant deficiencies in the formation of striated ⍺-actinin structures, noticeably reduced by day 12 of DMSO treatment, accompanied by little or no expression of Troponin-I (Fig 5E and 5G). Furthermore, we generated CRISPR-Cas9 P19CL6 clones with a knockout of Tab2, P19CL6^*Tab2−/−*^ (S9B and S9C Fig). Similar to TAK1 mutant cells, P19CL6^*Tab2−/−*^ cells exhibited severe delay in cardiomyogenesis, as indicated by reduced expression of GATA4 and the absence of striated sarcomeric patterns of Troponin I and ⍺-actinin (S9D–S9H Fig). Hence, mutations in *Tak1* and *Tab2* hinder cardiomyocyte differentiation.

To confirm the results for *Tak1* mutant cells, we exposed P19CL6 cells to TAK1 inhibitors, Takinib and 5ZO [54,55] throughout the differentiation protocol. In the presence of either inhibitor, P19CL6 cells displayed a significant reduction in GATA4-positive nuclei by day 7 of DMSO stimulation (Fig 5H and 5I) as well as a marked reduction in formation of well-defined striated sarcomeric patterns of Troponin I and ⍺-actinin as compared to vehicle controls, with the most prominent inhibition of cardiomyocyte formation observed with 5ZO (Fig 5J and 5K). These studies confirm the CRISPR-Cas9-mediated mutant results, highlighting the importance of TAK1 in cardiomyogenesis.

Finally, we investigated the role of TAK1 in coordinating downstream cardiogenic signaling events at the primary cilium during cardiomyogenesis. TAK1 is a well-established regulator of key signaling pathways, including c-Jun N-terminal kinase (JNK), p38 mitogen-activated protein kinase, and Nuclear Factor kappa-light-chain-enhancer of activated B cells—all of which play crucial roles in cardiac cell fate specification and cardiomyocyte maturation [56,57]. To address this, we examined the phosphorylation of JNK1/2 in P19CL6 cells during differentiation following TAK1 inhibition with 5ZO. IFM analysis revealed that in control cells, phosphorylated JNK1/2 predominantly localized to the ciliary base area, peaking at day 10 of DMSO treatment (Fig 5L and 5M). In contrast, 5ZO-treated cells exhibited consistently low levels of JNK1/2 phosphorylation at the ciliary base at day 0 of the experiment and throughout differentiation, with significantly reduced levels compared to controls (Fig 5L and 5M). Likewise, western blot analysis revealed reduced levels of phosphorylated Jnk1a/b in *tak1* mutants compared with *tak1*^*+/+*^ zebrafish larvae at 3 dpf (Fig 5N and 5O). Because TAK1 inhibition has previously been shown to decrease SMAD2/3 phosphorylation [58,59], we finally examined the effect of 5ZO on TGFB-1- and BMP2-induced phosphorylation of SMAD2/3 and SMAD1/5, respectively, in RPE-1 cells. These experiments demonstrate that TAK1 inhibition abolishes SMAD2/3 activation while leaving SMAD1/5 activation intact (S10A–S10D Fig), indicating that TAK1 differentially intersects with distinct branches of the TGFB superfamily signaling network. Together, these findings further support the primary cilium as a central hub for TAK1-mediated signaling within the non-canonical TGFB/BMP pathway and underscore its role in transmitting TAK1-dependent cues that regulate cardiogenesis, as depicted in our working model (Fig 5P).

## Discussion

Variants in *TAK1*, *TAB2* and *PRKACA* are associated with rare human multisystem disorders, which may include CHD and cardiomyopathy as part of the clinical spectrum [11–14,17,37]. Our analysis of exome sequencing data from a large CHD cohort, combined with in vivo and in vitro experiments support a significant role of both TAK1 and TAB2 in heart development and CHD. Importantly, we show that TAK1 operates in conjunction with TAB2 and PKA-Cα in non-canonical TGFB/BMP signaling at the primary cilium, and that FMD-associated mutations in TAK1 diminish its ciliary localization. These findings add to the growing evidence that underscores a significant role of cilia not only in cardiac left-right patterning but also in broader aspects of cardiac development and CHD [5,8].

In zebrafish *tak1* mutants, we observed several cardiac abnormalities, including increased myocardial cell proliferation. The delayed onset of these defects after heart tube formation suggests that early cardiac progenitor specification is largely intact, with disruptions emerging during later growth and differentiation. The underlying cellular mechanisms remain unclear. On the other hand, studies in P19.CL6 stem cells showed that TAK1 mutation or inhibition impairs early cardiomyogenesis, directly affecting initial differentiation in vitro. This discrepancy may reflect differences between in vivo and in vitro environments: the former includes cellular heterogeneity, tissue-level signaling, and compensatory mechanisms that can buffer early defects. Supporting a role for TAK1 in cardiomyocyte biology, transcriptome analysis of zebrafish *tak1* mutants revealed downregulation of key cardiac transcription factors and sarcomere genes. Nevertheless, the precise relationship between BMP/TGFB/TAK1 signaling and the observed cardiac phenotypes remains to be clarified.

In control cells, TAK1 function involved TAB2 recruitment, TAK1 phosphorylation and subsequent activation of downstream signaling via JNK1/2 at the primary cilium. Further substantiating a function of this signaling axis in cardiomyogenesis, in vitro cardiomyocyte differentiation is similarly obstructed in cells with knockout of TAB2. Based on these findings, we propose a model where the cilium takes center stage in coordination of cardiogenic signaling events where TAK1, in concert with other signaling pathways, controls intrinsic processes of cardiogenesis. This signaling crosstalk includes, but may not be limited to, the canonical branch of TGFB/BMP signaling and Hh signaling (Fig 5P). In support of this model, which emphasizes the interplay among ciliary pathway modules in regulating developmental signaling outcomes, TAK1 has been shown to bind and regulate the activity of R-SMADs [60], and upstream of TAK1, PKA-Cα downregulates Hh signaling by processing GLI2/3 transcription factors within the ciliary compartment [47]. Conversely, inhibiting GLI1 in the Hh pathway was shown to activate the TAK1-JNK1/2 signaling axis [61], introducing another layer of interaction between Hh and TAK1 signaling systems. We have also noted that TAK1 activation in a cell type-specific context is regulated by WNT5A [60,62], which modulates the length of primary cilia [63] and was proposed to operate via a ciliary TMEM67-ROR2 receptor complex in the planar cell polarity branch of WNT signaling [64]. Our discovery of TAK1 signaling in the primary cilium thus paves the way for future studies to unravel the complexities of developmental signaling networks and how the coordination of such networks may operate in a temporal manner to control cardiogenic events.

In addition to its potential role in cardiomyogenesis, our cardiac transcriptome analysis of zebrafish larvae indicates that TAK1 regulates a broad range of genes related to the structure and metabolism of the cardiac ECM. Although further studies are needed to dissect the specific changes in ECM components, this observation provides insight into some of the effects of TAK1 loss on cardiac development. Precise orchestration of ECM synthesis is crucial for establishment of the endocardial cushions (EC), the anlage of cardiac valves, which are cellularized by endothelial-to-mesenchymal transition (endoMT), a process regulated by primary cilia, of endocardial cells lining the EC [65,66]. Thus, ciliary TAK1 dysregulation of cardiac ECM is likely to explain part of the valvular phenotypes, which are observed in patients with *TAK1* and *TAB2* variants [11,67].

In support of this conclusion, TGFB/BMP signaling events critically regulate production and turnover of ECM across different contexts [68], and we speculate such events converge on TAK1, which influences both canonical and non-canonical TGFB superfamily signaling, cardiac ECM formation and, consequently, establishment of endocardial cushions. Moreover, ECM integrity impacts the bioavailability of TGFB/BMP ligands [68], and primary cilia themselves interact with ECM [69]. However, further investigations are required to fully understand how dysregulation of ciliary TAK1 manifests in valvular phenotypes. Our observation of a reduced number of Sox9-expressing mesenchymal cells in zebrafish *tab2* mutants, but not in *tak1* mutants, also suggests that TAB2 might play a TAK1-independent role in regulation of endoMT. Such a role is supported by our previous observation of prominent TAB2 expression within the EC [12] and could explain the higher prevalence of valve defects in patients with *TAB2* defects [11].

In both *tak1* and *tab2* mutant zebrafish larvae, we observed significantly reduced trabeculation. Although a modest increase in heart rate was detected in the mutants, its relationship to the trabeculation phenotype remains unclear. The elevated heart rate observed at later stages (5 dpf) may represent a compensatory response to impaired cardiac function rather than a driver of proper morphogenesis [70]. Cardiac trabeculation is regulated by a delicate balance between synthesis and degradation of the ECM [71]. In this regard, the pronounced dysregulation of ECM genes in *tak1* mutants provides a plausible molecular explanation for the reduced trabeculation we observed in *tak1* and *tab2* mutants. Considering the many similarities between our zebrafish mutants and the clinical phenotype in patients, the trabeculation defects observed in *tak1* mutants warrant increased attention on trabeculation defects among patients with severe *TAK1* variants.

Finally, extracardiac defects are observed among FMD, CSCFS, and THS patients [11,13,14,67]. Since *tak1* and *tab2* mutants were lethal prior to adult stage, it was not possible to investigate extracardiac defects in detail, but we noted some resemblances with the human syndromes, in the form of craniofacial and pectoral fin anomalies. Thus, the *tak1* and *tab2* mutants appear valid as animal models for FMD, CSCFS, and THS, offering the potential for further advancing our understanding of disease pathomechanisms and as screening models for development of small-molecule therapeutics.

In conclusion, our results identify ciliary coordination of TAK1 signaling as an essential mechanism in cardiac development and provide novel insight into the pathomechanisms of human syndromes related to TAK1, TAB2 and PKA-Cα.

## Supporting information

S1 Fig Burden of pathogenic variants in sCHD patients and CRISPR-Cas9 editing in zebrafish *tak1* and *tab2.* **(A)** Distribution of CADD scores for variants identified in all CHD patients (aCHD) and controls. **(B)** Distribution of CADD scores for variants identified in sCHD patients and controls. **(C)** Burden of pathogenic variants in sCHD. **(D, E)** Sanger sequencing chromatograms displaying *tak1*^*−/−*^ (D) and *tab2*^*−/−*^ (E) mutant zebrafish sequences in comparison to *tak1*^*+/+*^ and *tab2*^*+/+*^ WT sequences. Dashed boxes mark deleted nucleotides in the mutant zebrafish. **(F)** Corresponding protein sequences after mutations in zebrafish *tak1* and *tab2*. Dark gray indicates added amino acids, while light gray highlights untranslated region. **(G)** validation of Tak1 knockout in *tak1*^*−/−*^ mutant zebrafish by western blotting analysis. Source data are available in S2 Data.
(TIFF)

S2 Fig Cardiac phenotype analysis of *tak1* mutants. **(A)** In situ hybridization with *nkx2.5* and *mef2cb* RNA probes on 14 hpf *tak1*^*+/+*^ and *tak1*^*−/−*^ embryos. Scale bar, 0.2 mm. **(B)** Gross morphology of 5 dpf *tak1*^*+/+*^ and *tak1*^*−/−*^ zebrafish larvae highlighting pericardial edema (black arrow) and protruding mouth (open arrow). Scale bar, 0.5 mm. **(C)** IFM with β-catenin (green) and Mef2a/c (red) antibodies in 3 dpf *tak1*^*+/+*^/*tak1*^*+/−*^ and *tak1*^*−/−*^ extracted zebrafish hearts. Scale bars, 20 μm. **(D)** Quantification of cell size in atrium and ventricle of 3 dpf *tak1*^*+/+*^/*tak1*^*+/−*^ and *tak1*^*−/−*^ zebrafish hearts. Each dot represents an average of 10 cells in a larvae (*n* = 3). **(E)** Wholemount IFM with α-actinin (green) antibody marking z-discs in the 3 dpf *tak1*^*+/+*^/*tak1*^*+/−*^ and *tak1*^*−/−*^ zebrafish hearts. Scale bars, 20 μm. **(F)** Quantification of z-disc distance in the atrium and the ventricle of 3 dpf *tak1*^*+/+*^/*tak1*^*+/−*^ (*n* = 5) and *tak1*^*−/−*^ (*n* = 6) zebrafish hearts. Each dot represents an average of 10 sarcomere units in a larvae. **(G)** IFM with Sox9 antibody (red) alone (upper) or in combination with Alcama (lower) in 5 dpf *tak1*^*+/+*^ and *tak1*^*−/−*^ extracted zebrafish hearts. Scale bars, 20 μm. **(H)** Quantification of number of mesenchymal cells (Sox9 positive cells) in AV valves of *tak1*^*+/+*^ and *tak1*^*−/−*^ zebrafish hearts at 3 dpf (*n* = 11, 12) and 5 dpf (*n* = 16, 13). **(I)** Acridine orange staining (green) of 3 dpf *tak1*^*+/+*^ and *tak1*^*−/−*^ zebrafish showing apoptotic cells. Scale bars, 0.1 mm. **(J)** Relative expression of *bcl2* (left) and *baxa* (right) in 3 dpf *tak1*^*+/+*^ (*n* = 10) and *tak1*^*−/−*^ (*n* = 9) extracted zebrafish hearts. *t* test, *: *P* < 0.05, ns: not significant. Nuclei were stained with DAPI (blue). Source data are available in S2 Data.
(TIFF)

S3 Fig Phenotypes of *tab2* mutants. **(A)** Gross morphology of 5 dpf *tab2*^*+/+*^ and *tab2*^*−/−*^ zebrafish larvae highlighting pericardial edema (black arrow) and protruding mouth (open arrow). Scale bars, 0.2 mm. **(B)** Survival of *tab2*^*+/+*^ (*n* = 12), *tab2*^*+/−*^ (*n* = 27) and *tab2*^*−/−*^ (*n* = 10) zebrafish larvae. **(C)** Upper panels: wholemount IFM of transgenic *Tg(myl7:GFP) tab2*^*+/+*^ and *tab2*^*−/−*^ zebrafish hearts with GFP (green) and Myh6 (red) antibodies at 5 dpf. A: atrium, V: ventricle. Lower panels: wholemount IFM of transgenic *Tg(myl7:GFP) tab2*^*+/+*^ and *tab2*^*−/−*^ zebrafish hearts with GFP antibody (green) at 6 dpf. Nuclei were stained with DAPI (blue). Ventricular trabeculae are indicated with arrows and trabeculation defects with an arrow head. Scale bars, 20 μm. **(D)** Quantification of normalized atrium area, ventricle area (*n* = 24, 38), ejection fraction (%), fractional shortening (%) (*n* = 23, 21), heart rate (*n* = 36, 36)at 5 dpf and number of trabecular ridges (*n* = 4, 5) at 6 dpf. **(E)** IFM of cardiomyocyte nuclei and proliferating cells, using anti-Mef2a/c (red) and anti-Pcna (green) antibodies, respectively in 5 dpf *tab2*^*+/+*^ and *tab2*^*−/−*^ zebrafish heart extracts (DAPI, blue). Scale bars, 20 μm. **(F)** Quantification of number of cardiomyocytes (Mef2a/c-positive cells) in atrium and ventricle of 5 dpf *tab2*^*+/+*^ (*n* = 9) and *tab2*^*−/−*^ (*n* = 9) zebrafish hearts. **(G)** Quantification of cell proliferation index in atrium and ventricle of 5 dpf *tab2*^*+/+*^ (*n* = 9) and *tab2*^*−/−*^ (*n* = 9) zebrafish hearts. **(H)** IFM with Sox9 antibody (red) alone or in combination with Alcama in 5 dpf *tab2*^*+/+*^ and *tab2*^*−/−*^ extracted zebrafish hearts. Nuclei were stained with DAPI (blue). Scale bars, 20 μm. **(I)** Quantification of number of mesenchymal cells (Sox9-positive cells) in AV valves of *tab2*^*+/+*^ (*n* = 19) and *tab2*^*−/−*^ (*n* = 14) zebrafish hearts at 5 dpf. **(J)** Bright-field images of *tab2*^*+/+*^*/tab2*^*+/−*^ (upper panels) and *tab2*^*−/−*^ (lower panels) larvae showing (i) lateral views of cartilage stained with Alcian blue at 5 dpf. (ii) Meckel’s-palatoquadrate (M-PQ) angle measurements of cartilage. Black lines show the measured angle in larvae. (iii) Measurement of pectoral fin length. Black line shows measured fin length. (iv) Eye distance measurement at 7 dpf. Black line shows the measured eye distance in larvae. Scale bars, 0.1 mm. (K) Quantification of measurements obtained from i (*n* = 29, 20), ii (*n* = 27, 22), iii (*n* = 16, 10). *t* test, **: *P* < 0.01, ***: *P* < 0.001, ****: *P* < 0.0001, ns: not significant. Source data are available in S2 Data.
(TIFF)

S4 Fig OFT in *tak1* and *tab2* mutants. **(A, B)** Wholemount IFM using a GFP antibody (green) combined with in situ hybridization for *elnb* mRNA (red) in 3 dpf *tak1*^*+/+*^ and *tak1*^*−/−*^ (A); and 5 dpf *tab2*^*+/+*^ and *tab2*^*−/−*^ (B) zebrafish Scale bar: 50 μm. Quantification of the OFT area marked by *elnb* is shown on the right of each corresponding panel. Statistical analysis was performed via *t* test, **: *P* < 0.01, ****: *P* < 0.0001. Source data are available in S2 Data.
(TIFF)

S5 Fig Pharmacological inhibition of Tak1 in zebrafish. **(A)** Gross morphology of 3 dpf WT (AB strain) zebrafish larvae treated with 1% DMSO and 10 μM Takinib, with arrowhead pointing to pericardial edema (top left) Scale bar: 500 μm. Representative images of 3 dpf *Tg(myl7:GFP)* hearts under the same treatment conditions (top right) Scale bar: 100 μm. Quantification of normalized atrium and ventricle areas in *Tg(myl7:GFP)* hearts (bottom left) (*n* = 41, 41). **(B)** Gross morphology of 3 dpf WT (AB strain) zebrafish larvae treated with 1% DMSO and 5 μM 5ZO, with arrowhead pointing to pericardial edema (top left) Scale bar: 500 μm. Representative images of 3 dpf *Tg(myl7:GFP)* hearts under the same treatment conditions (top right) Scale bar: 100 μm. Quantification of normalized atrium and ventricle areas in *Tg(myl7:GFP)* hearts (bottom left) (*n* = 21, 21). **(C)** Wholemount IFM using a GFP antibody (green) in 6 dpf *Tg(myl7:GFP)* hearts treated with 1% DMSO and 5 μM 5ZO. Scale bar: 20 μm. Quantification of the number of trabecular ridges is shown (*n* = 10, 10). *t* test, ***: *P* < 0.001, ****: *P* < 0.0001, ns: not significant. Source data are available in S2 Data.
(TIFF)

S6 Fig RNA sequencing and RT-qPCR. **(A)** Enrichment of known CHD genes within 474 down- and 712 upregulated genes (blue and red circles, respectively). Enrichment is shown as representation factor (RF). A hypergeometric distribution was used to test the significance. MmCHD: a list of 832 genes causing heart defects in mouse models [39]. HsCHD: a list of 377 genes causing CHD in human patients [38]. **(B, C)** RT-qPCR of differentially expressed genes found in RNA-seq between (B) *tak1*^*+/+*^ and *tak1*^*−/−*^ extracted hearts (RT-qPCR for *tak1* was performed on the whole larvae) at 3 dpf (*n* = 3–6) and (C) *tab2*^*+/+*^ and *tab2*^*−/−*^ extracted hearts at 5 dpf (*n* = 3). The samples used for RT-qPCR verification were independent from the samples used for RNA-seq. *t* test, *:*P* < 0.05, **: *P* < 0.01, ****: *P* < 0.0001. **(D)** Enrichment of upregulated genes in the expression signature of 15 cell-types in 6.5–7 PCW human developing hearts [40]. Enrichment is shown as representation factor (RF). A hypergeometric distribution was used to test the significance. Non-significant enrichment is marked with gray circles. Source data are available in S2 Data.
(TIFF)

S7 Fig Zebrafish larvae extracardiac cilia and characterization of day 7 gastruloids. **(A)** IFM of extracardiac tissue in 3 dpf WT zebrafish larvae stained with Tak1 (green) and acetylated tubulin antibody (Ac-tub) (magenta). Arrow points to the cilium (insert). Scale bar, 100 µm. **(B)** Representative IFM image indicating the expression of cardiac junction protein N-Cadherin (orange) in NKX2.5 positive cells/cardiomyocytes (green) and the epithelial cadherin, E-Cadherin (magenta) in intestinal epithelium of day 7 gastruloids. Small image scale bar, 40 µm, large image scale bar, 20 µm. **(C, D)** Representative IFM images of day 7 gastruloids showing HCN4 (red, marks FHF) (C) and Troponin-I (red) (D) in NKX2.5 (green) positive cells. **(E)** Localization of TAK1 in Day 7 gastruloid, white boxes indicate ROIs. NKX2.5 (green) positive cardiac progenitor cells and ARL13B (white) stained primary cilia. TAK1 (green) is observed at primary cilia of day 7 gastruloids shown with both merged and shifted images. Colored arrows in shifted image denote the different channels. Large image scale bars, 10 μm.
(TIF)

S8 Fig TAK1 localizes to the ciliary basal region and transiently along the axoneme. Representative IFM images of RPE-1 cells showing TAK1 (green) at the primary cilium stained with ARL13B (blue) together with Centrin-2 (red) **(A)**, CEP164 (red) **(B)** and IFT88 (red). **(C)**. Closed arrow points to the cilium, open arrow points to the distal appendage of the basal body. * Denotes the ciliary base. Scale bars: 2 µm.
(TIF)

S9 Fig CRISPR-Cas9 editing in P19CL6 cells and differentiation phenotype in *Tab2*^−/−^ cells. **(A, B)** Graphical illustration of *Tak1* CRISPR mutant clone P19CL6^*Tak1Δ85/Δ85*^ (A) and *Tab2* CRISPR mutant clone P19CL6^*Tab2−/−*^ (B) showing deletions (black) and predicted premature stop codons (red lines). The deletion in P19.CL6^*Tak1Δ85/Δ85*^ cause aberrant splicing (skipping of exon 2), which results in a deletion (p.Val41-Glu77del). **(C)** Western blot analysis of P19CL6^WT^, P19CL6^*Tab2−/−*^ and P19CL6^*Tak1Δ85/Δ85*^ cells using antibodies against TAK1, TAB2 and DCTN1. **(D)** Western blot analysis of DMSO-treated P19.CL6^*Tab2−/−*^ cells using antibodies against SOX2, GATA4 and DCTN1. **(E)** Representative IFM images of expression of OCT3/4 (green) and GATA4 (red) at day 7 of DMSO stimulation in P19CL6^*Tab2−/−*^ cells. Scale bar, 10 μm. **(F)** Percentages of GATA4-positive nuclei in P19CL6^WT^ and P19CL6^*Tab2−/−*^ at day 7 (*n* = 3, WT: 1,005 nuclei, TAB2^−/−^: 915 nuclei), ****P < 0.0001 (*t* test). **(G)** α-actinin (green) and Troponin-I (pseudocolored magenta) at day 12 of DMSO stimulation in P19CL6^*Tab2−/−*^ cells. Scale bar, 20 μm. **(H)** Relative levels of α-actinin in P19.CL6^WT^ and P19.CL6^*Tab2−/−*^cells at day 12 (*n* = 3, WT: 18 areas, TAB2^−/−^: 18 areas). ****P < 0.0001 (*t* test). Source data are available in S2 Data.
(TIF)

S10 Fig Effect of TAK1 inhibition on phosphorylation of R-SMADs. Western blot analysis of serum-starved RPE-1 cells treated with 500 nM 5ZO or corresponding volume DMSO and stimulated with TGFB-1 **(A)** or BMP2 **(C)** and quantified in **(B)** and **(D)**, respectively. *t* test. ***: *P* > 0.001, *: *P* > 0.05, ns: not significant. Source data are available in S2 Data.
(TIF)

S1 Table Rare variants identified using exome sequencing data from 3,876 CHD cases and 45,082 controls. (XLSX)

S2 Table Up and downregulated genes in hearts dissected from 3 dpf tak1^−/−^ zebrafish. (XLSX)

S3 Table Primary antibodies. (XLSX)

S4 Table Secondary antibodies. (XLSX)

S5 Table Primers. (XLSX)

S1 Data Source data for Figs 1–5. An Excel spreadsheet containing the underlying numerical data for Figs 1A–1C, 1E, 1G, 1I, 1K, 2A–2D, 3F, 3H, 3J, 4H, 4I, 5B, 5D, 5F, 5G, 5I, 5K, and 5M.
(XLSX)

S2 Data Source data for S1–S10 Figs. An Excel spreadsheet containing the underlying numerical data for S1A–S1C, S2D, S2F, S2H, S2J, S3B, S3D, S3F, S3G, S3I, S3K, S4A, S4B, S5A, S5B, S5C, S6A–S6D, S9F, S9H, S10B, and S10D Figs.
(XLSX)

S1 Raw Images Raw images of all blots and gels. (PDF)

## Abbreviations

AV: atrioventricular
CADD: Combined Annotation Dependent Depletion
CAFD: cardioacrofacial dysplasia
CHD: congenital heart disease
CSCFS: cardiospondylocarpofacial syndrome
DIG: Digoxigenin
ECM: extracellular matrix
EZRC: European Zebrafish Resource Center
FBS: fetal bovine serum
FMD: frontometaphyseal dysplasia
GO: Gene ontology
HCOP: HGNC Comparison of Orthology Predictions
IFM: Immunofluorescence microscopy
mESCs: Mouse embryonic stem cells
nsCHD: non-syndromic CHD
OFT: outflow tract
PAVs: Protein-altering variants
PBS: phosphate-buffered saline
PFA: paraformaldehyde
PTU: 1-phenyl-2-thiourea
ROI: region of interest
sCHD: syndromic CHD
SYNs: synonymous variants
TAB2: TAK1 Binding Protein 2
TAK1: Transforming Growth Factor-Beta-Activated Kinase 1
THS: TAB2 haploinsufficiency syndrome
