# Supplementary material for: TAK1 operates at the primary cilium in non-canonical TGFB/BMP signaling to control heart development
Source: PLoS Biol. 2026 Aug 4;24(8):e3003902. doi: 10.1371/journal.pbio.3003902 (PMC13436766; doi:10.1371/journal.pbio.3003902)

Suppl Figure 1G

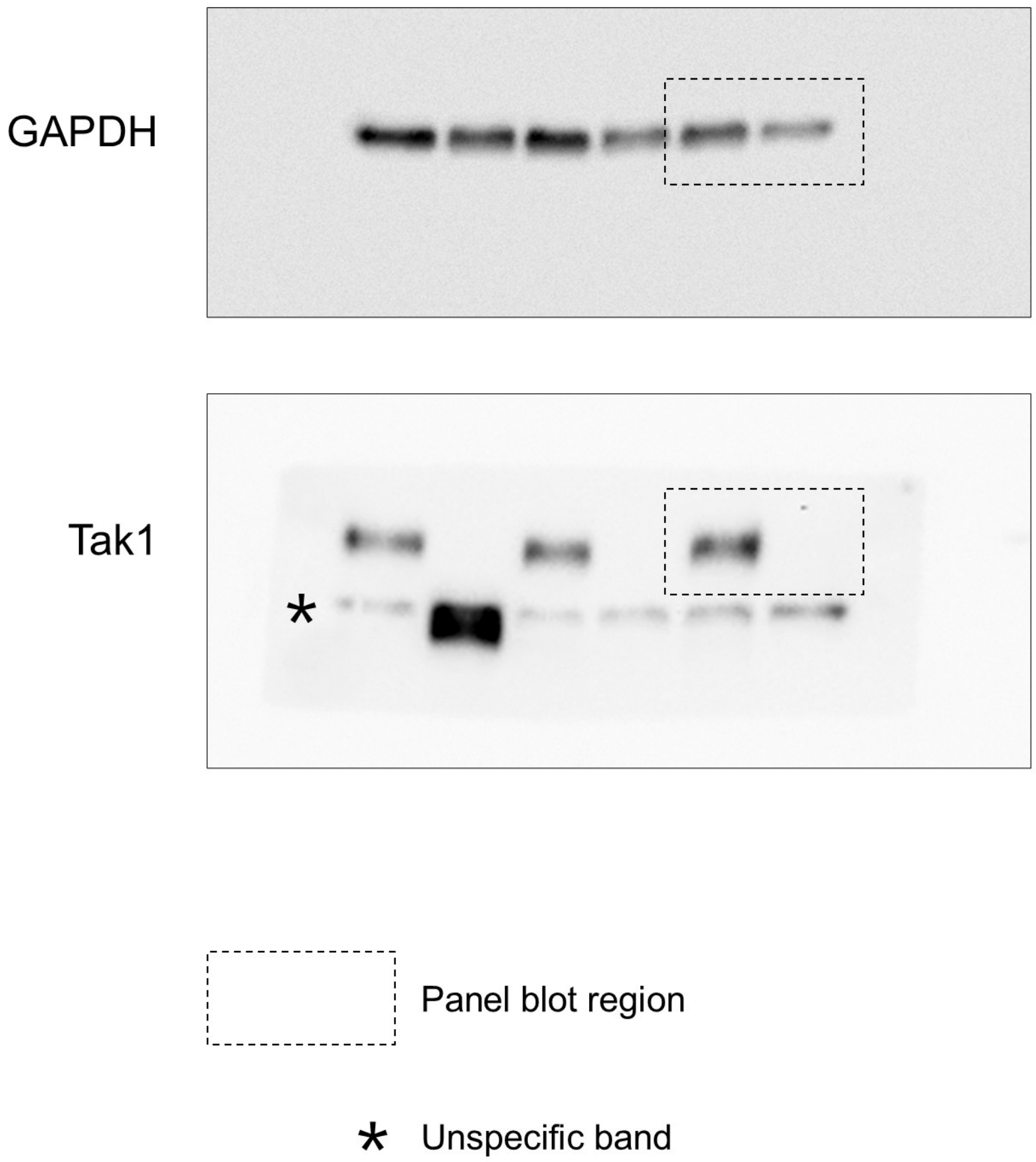

Figure 3E

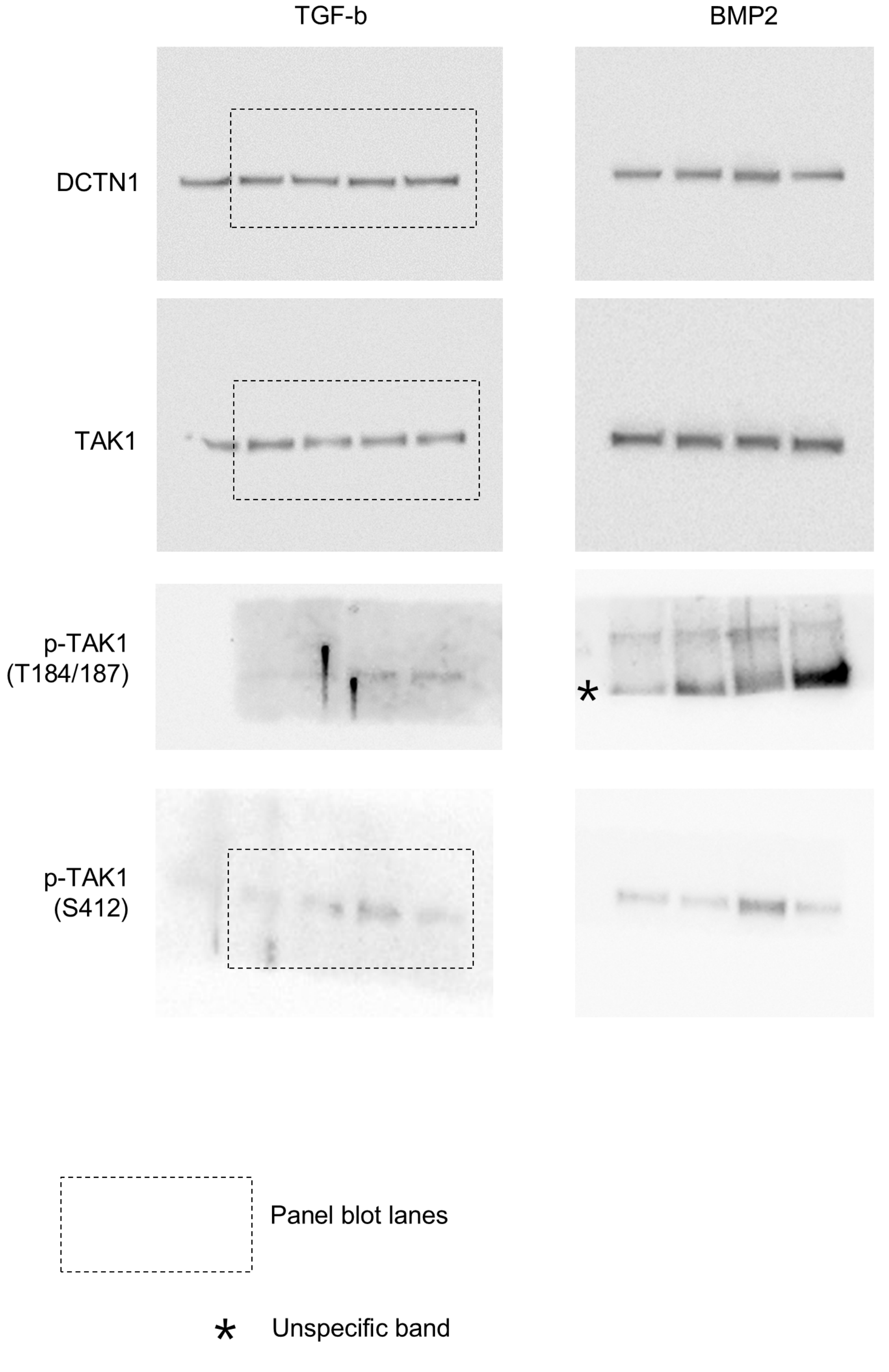
TGF-b
BMP2
DCTN1
TAK1
p-TAK1
(T184/187)
*
p-TAK1
(S412)
Panel blot lanes
* Unspecific band

Figure 3K

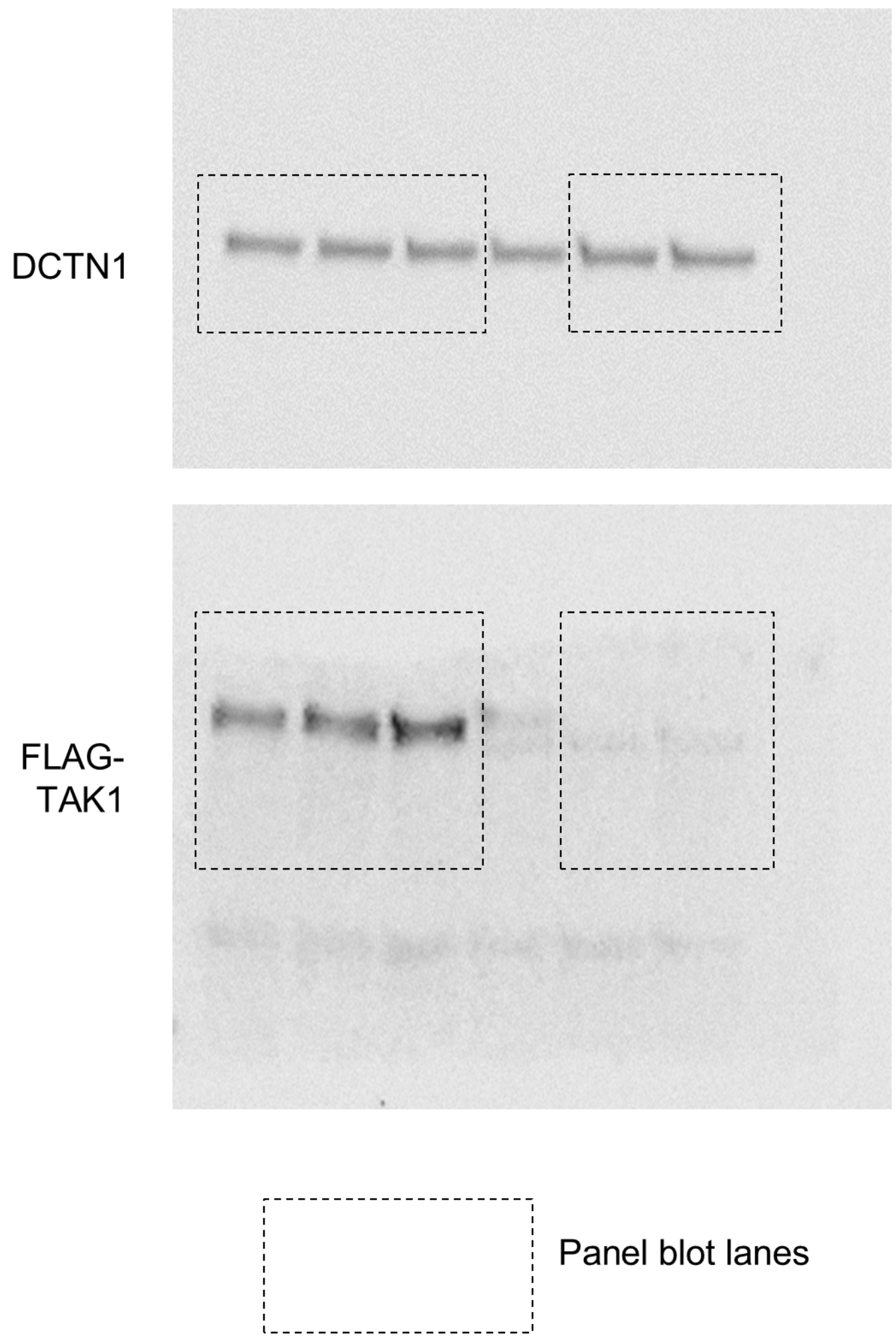

Figure 4B

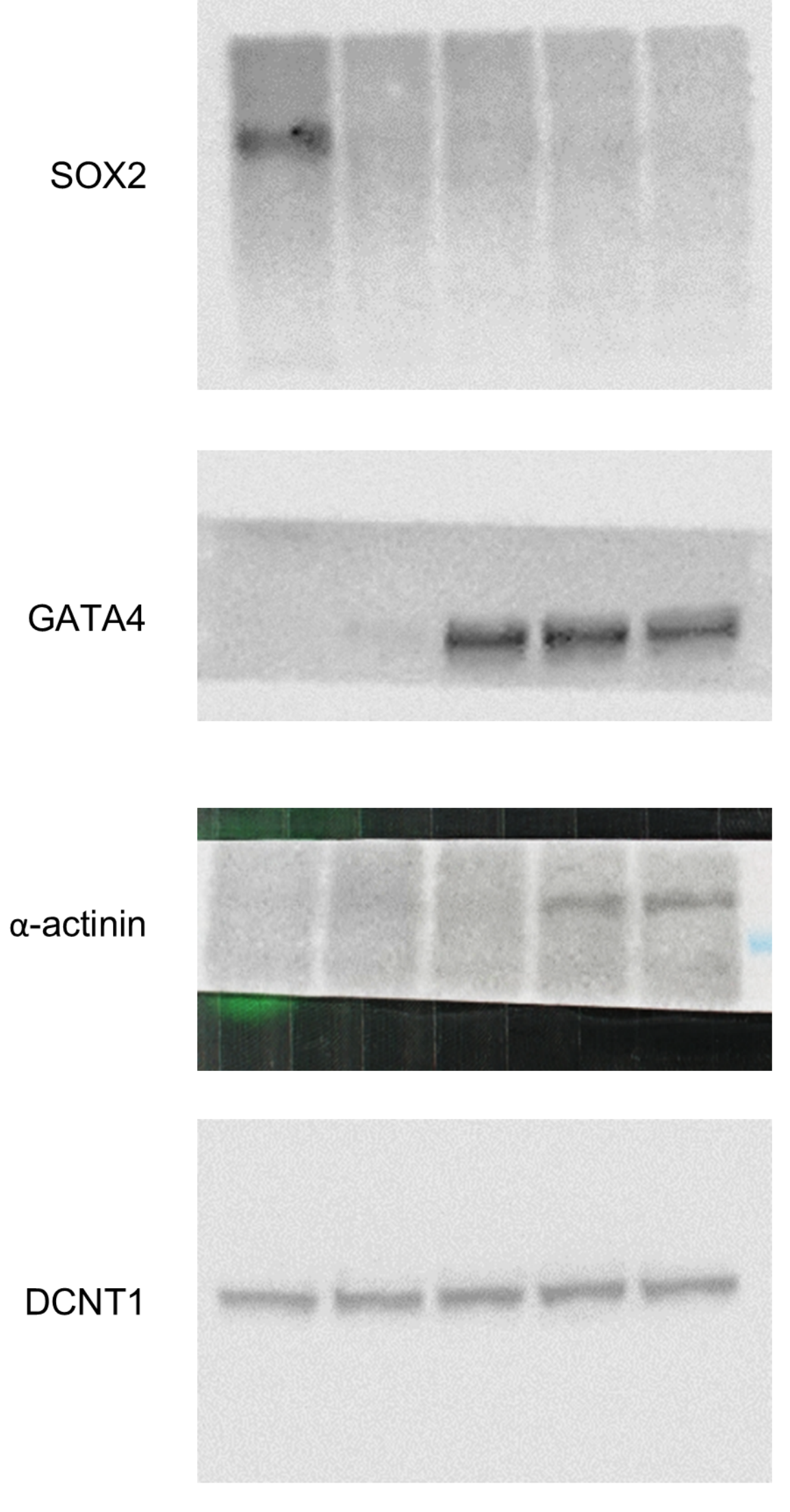

Figure 5A and 5C

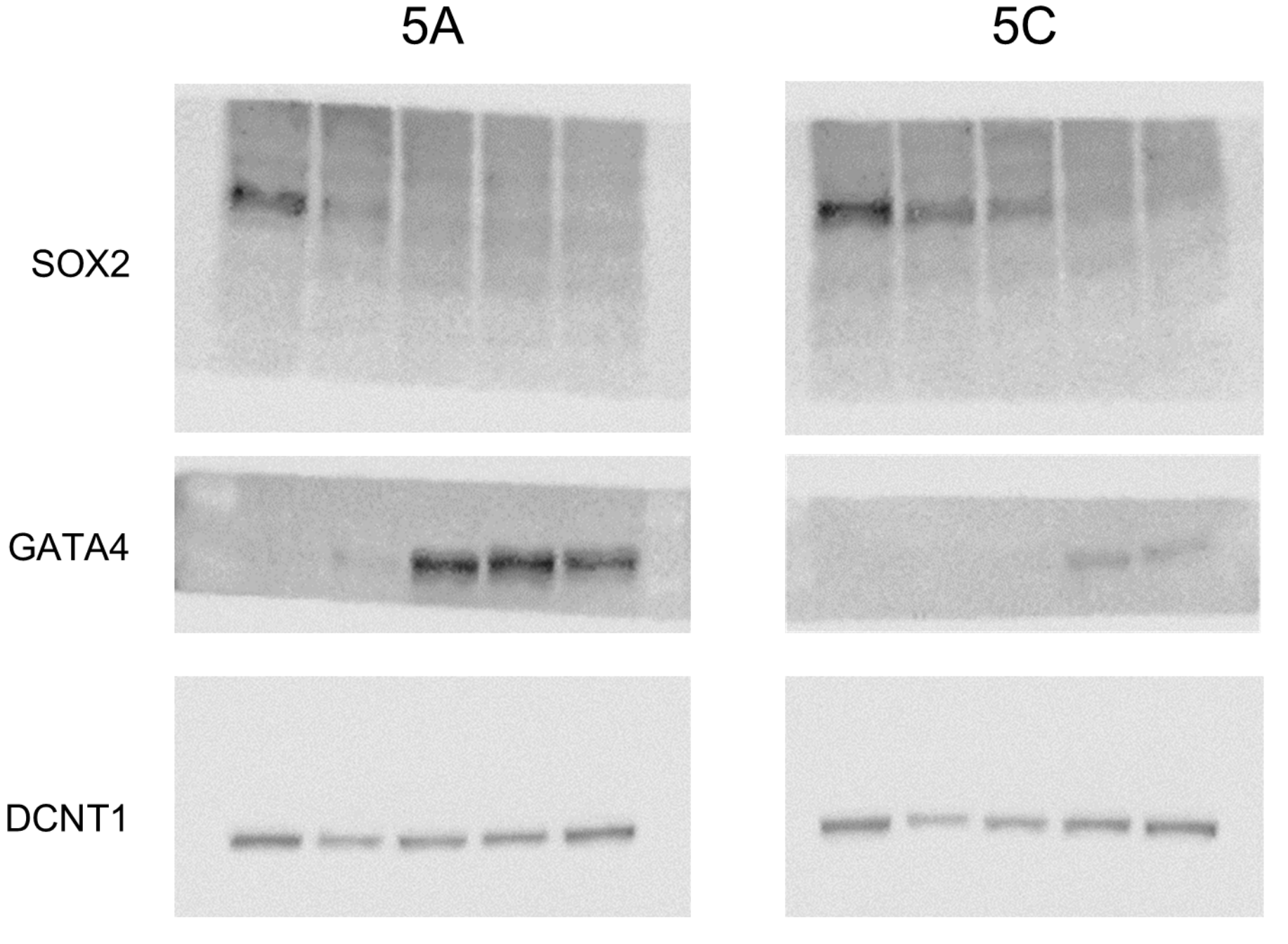

Figure 5N

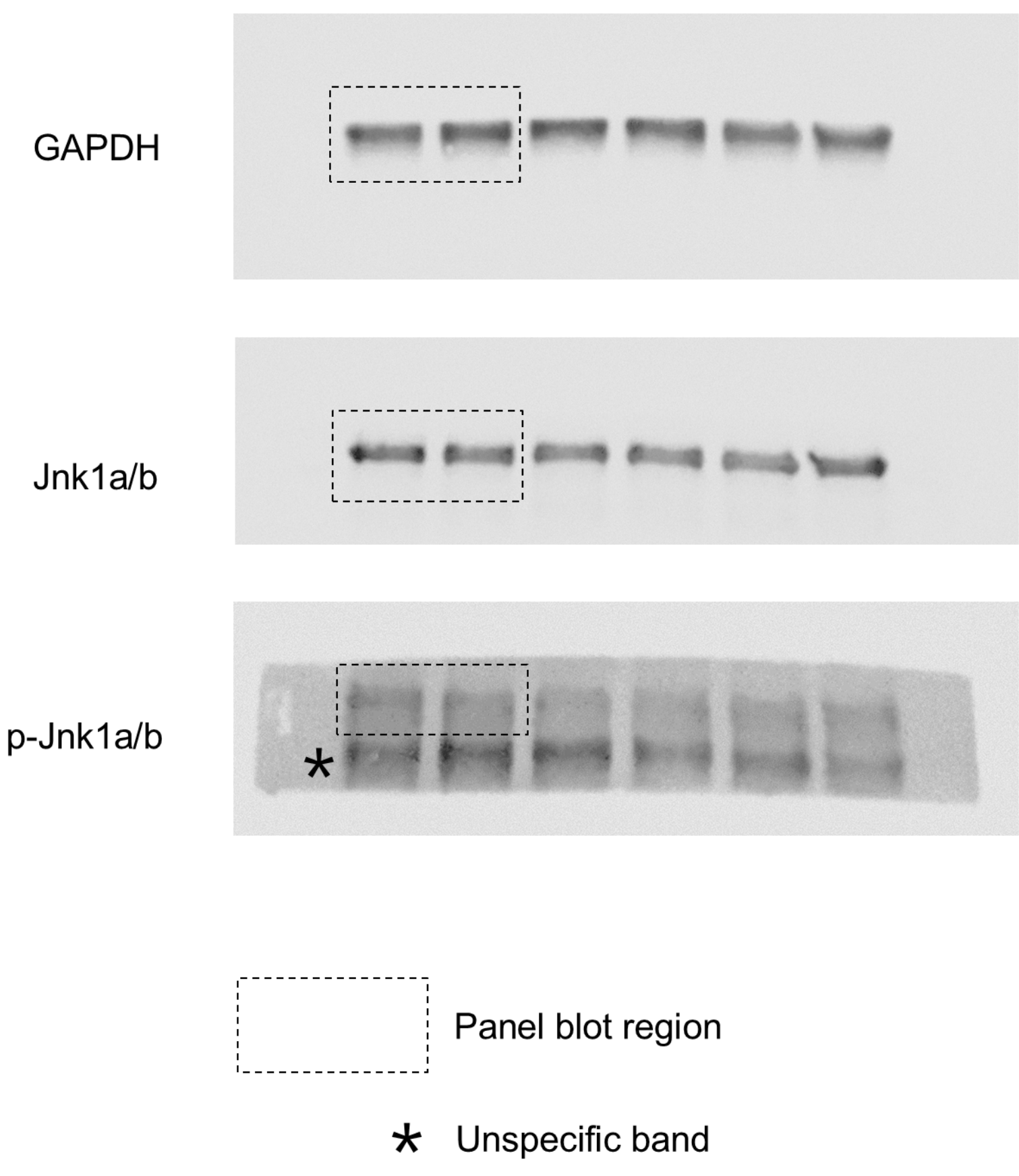

Suppl Figure 9C and 9D

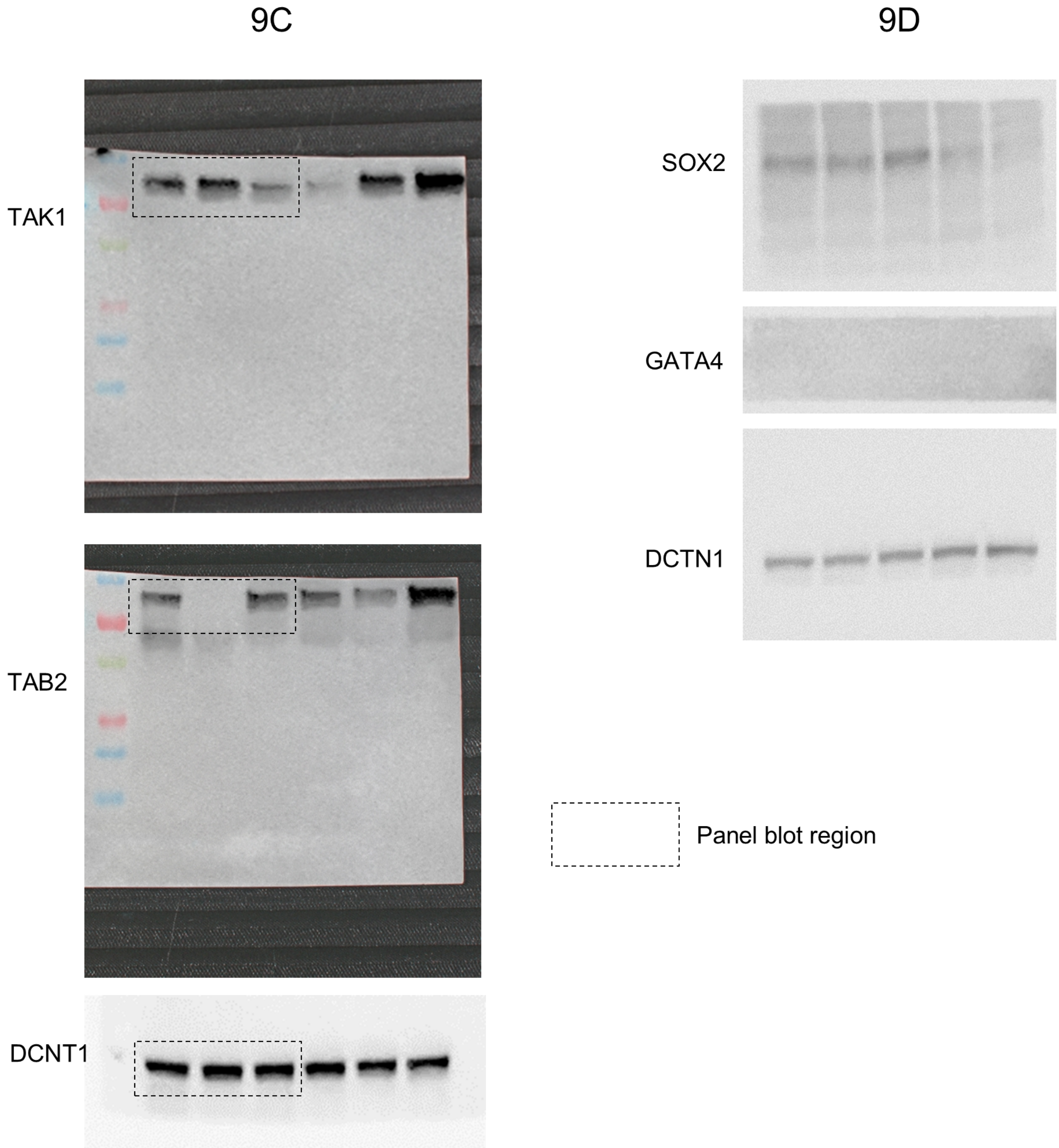

Suppl Figure 10A and 10C

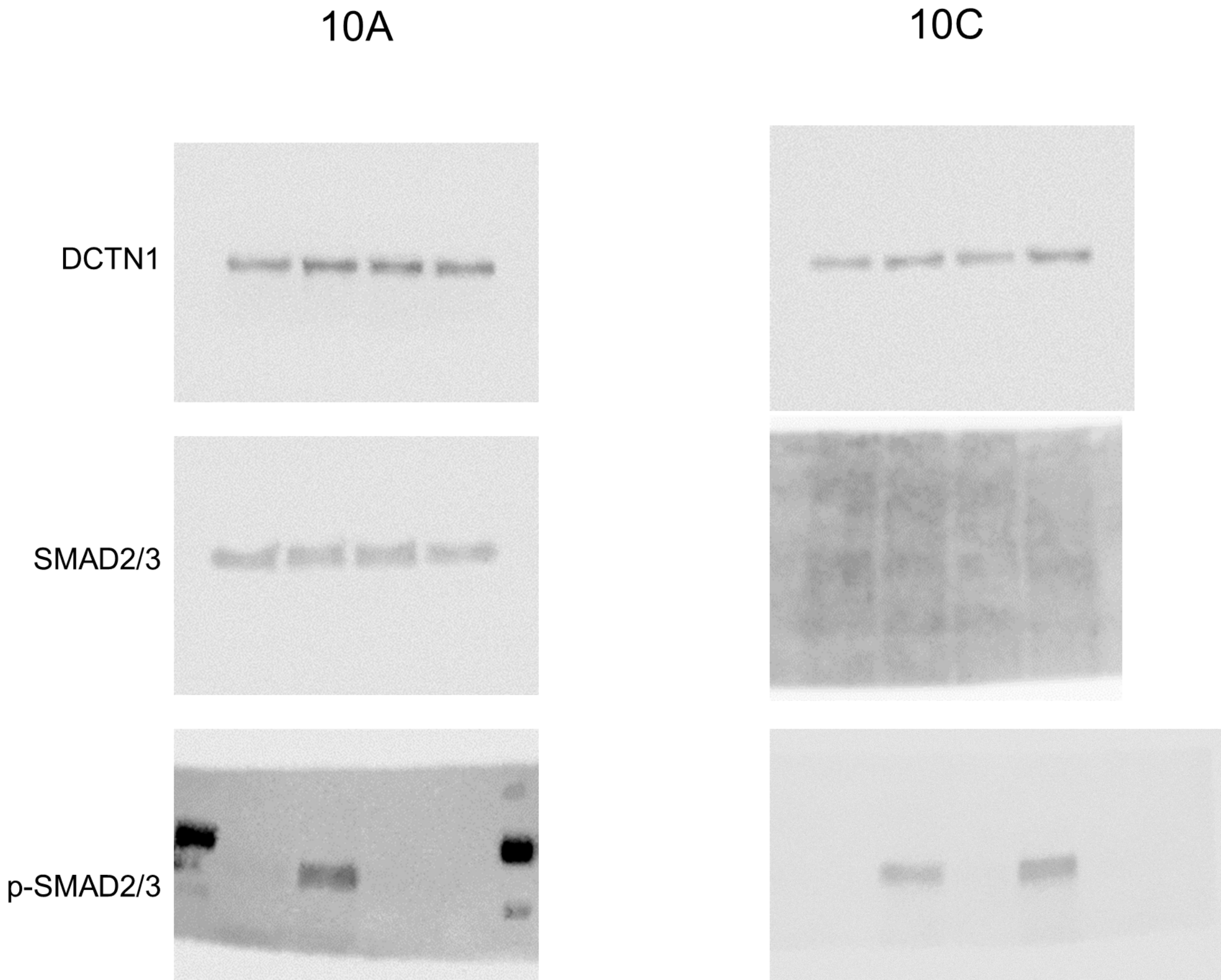

Supplement: Supplementary file 1 [file pbio.3003902.s018.pdf]
