# Supplementary material for: TAK1 operates at the primary cilium in non-canonical TGFB/BMP signaling to control heart development
Source: PLoS Biol. 2026 Aug 4;24(8):e3003902. doi: 10.1371/journal.pbio.3003902 (PMC13436766; doi:10.1371/journal.pbio.3003902)
Supplement: Supplementary file 3 [file pbio.3003902.s020.docx]

We sincerely thank the reviewers for their insightful and constructive comments, which have significantly improved our manuscript.

**Point by point responses to the reviewers:**

**Reviewer 1:**

In the manuscript from Doganli et al, they explore the requirement of TAK1 in cardiomyogenesis and its potential mechanism of action. Based on exome sequencing data that implicated TAK1 and its upstream regulator TAB2 as a risk factor in syndromic CHD, the authors generated zebrafish mutants lacking tak1 and tab2 which exhibited an array of cardiac developmental defects as well as craniofacial and limb defects. The zebrafish mutant models utilized in this study are said to not only recapitulate many of the cardiac and extracardiac defects associated with conditions such as FMD and CSCF, which can be caused by rare variants of TAK1. RNA sequencing of tak1 mutant hearts showed that loss of tak1 is associated with mis-regulation of cardiac transcription factors, sarcomeric proteins, and cardiac ECM proteins. The authors utilized complementary in vitro cell culture model systems to reveal that TAK1 and its signaling partners TAB2 and PKC-Cα localize to the primary cilium of differentiating cardiomyocytes. Consistent with their previous findings, the authors also demonstrated that inhibition of TAK1 function hindered cardiomyocyte differentiation in vitro. JNK1/2 phosphorylation at the base of cilia in these cells was dependent upon TAK1 activation, which they state must function through non-canonical TGFB signaling. The authors conclude that this research provides mechanistic insight into the TAK1-TAB2 signaling and sheds new light on its role in cardiomyocyte development. Their finding that pathogenic variants of TAK1 have defective ciliary localization may have clinical interest for associated syndromes with TAK1 dysfunction and cardiac and craniofacial defects. Although there are strengths to the manuscript, there are numerous concerns that the authors need to address. In particular, a significant amount of the presented data are not convincing and do not support the authors' conclusions.

Major issues:

1. The use of morpholinos to confirm phenotypes is a significant weakness. The morphant embryos shown in Fig S4 don't look anything like the mutants and display classical non-specific defects found with some morpholinos, such as smaller, curved body axis, smaller heads, and ventricular edema in the head. mRNA rescue is not an acceptable method to show specificity for morpholinos as it can lead to false positives (Stainier et al, 2017). An appropriate method of verification would be to have generated independent mutant alleles to show that they have the same defects. Were additional alleles of these genes generated?

We acknowledge the reviewer’s concerns regarding morpholino specificity and have therefore removed these data from the revised manuscript.

Subsequent to initiating experiments with the current *tak1* mutant, we identified an additional *tak1* allele carrying a 4 bp deletion. This line was bred only twice to assess whether the resulting phenotype, including gross morphological defects and pericardial edema, was consistent with that observed in the primary mutant line. The phenotypic consistency was confirmed. However, the line was discontinued due to the associated maintenance costs.

In addition, we performed pharmacological inhibition experiments using two independent Tak1 inhibitors (Takinib, (5Z)-7-oxozeaenol (5ZO)). Notably, inhibition of Tak1 produced cardiac phenotypes closely comparable to those observed in the *tak1* mutant zebrafish (Fig S5), which strengthens the causal link between Tak1 function and the observed defects.

2. Many of the phenotypes of the tak1 and tab2 mutant zebrafish (jaw and valve) are not the same and equivalent experiments were not performed, such as cardiomyocyte quantification. The jaws of the tak1 mutants (Fig. 1d) and tab2 mutants (Fig. S3a) look nothing alike. The difference between the mutants should be acknowledged appropriately in the manuscript and parallel experiments should be performed for these mutants for all experiments.

We understand the reviewer’s confusion regarding the jaw morphologies of the *tak1* and *tab2* mutants. Fig 1D and Fig S3A represent different developmental stages, 3 dpf (*tak1* mutants) and 5 dpf (*tab2* mutants) respectively. The jaw defect becomes prominently detectable by 5 dpf in *tak1* mutants, just as seen in *tab2* mutants. We have now included a representative image of *tak1* mutants at 5 dpf to better illustrate the observed phenotype (Fig S2B). Therefore, Alcian blue staining was conducted at 5 dpf in both cases to allow for consistent assessment of craniofacial cartilage morphology. We have now clarified the staging in all of the figure panels and also included a lateral image of Alcian blue staining in *tak1* mutants at 5 dpf (Fig 1J).

We indeed observe differences in valve defects between *tab2* and *tak1* mutants, which are consistent with patient data, where *TAB2* mutations are associated with more pronounced valve abnormalities. This point has now been incorporated into the discussion section.

We have further assessed and included cell number and proliferation assays in cardiac chambers for *tab2* mutants (Fig S3E-G).

3. It is not clear when the authors begin to see the developmental defects in the hearts of the tak1 and tab2 mutants. For instance at what stage can an increase number of atrial cardiomyocytes be observed?

We thank the reviewer for this important comment. We examined heart development beginning at early stages, including the cardiac progenitor stage; however, no obvious defects were detected at these early time points. We have now included this data in the manuscript (Fig S2A). In both mutants, cardiac abnormalities first become morphologically detectable around 2 dpf in a subset of embryos. The phenotypes become fully penetrant at later stages, specifically 3 dpf in *tak1* mutants and 5 dpf in *tab2* mutants. We have now clarified the timing of phenotype onset in the revised manuscript.

At 24 hpf no apparent morphological phenotype is visible, which makes it difficult to distinguish homozygous mutants from embryos derived from heterozygous crosses at this stage. Consequently, analyses such as cardiomyocyte quantification, which were performed on tens of pooled, isolated hearts (as whole-mount imaging did not provide sufficient resolution), become technically challenging as they would require linking imaging data to the genotypes of individual embryos, which is not feasible once samples are pooled.

4. Many of the cardiac defects seem to be contradictory to loss of trabeculation. Increased heart rate would be expected to increase blood flow and shear stress, which would promote trabeculation.

We thank the reviewer for raising this important point. While increased heart rate can indeed elevate blood flow and shear stress, factors typically associated with promoting trabeculation, our data suggest that the elevated heart rate in this model likely reflects a compensatory response to impaired cardiac function rather than increased effective flow. High heart rate in the context of structural defects (e.g., reduced contractility or poor chamber morphology) may not result in sufficient shear stress at the endocardium to support normal trabeculation. Additionally, the disruption of key signaling pathways may impair the endocardial-myocardial interactions required for trabeculation, overriding hemodynamic cues (Qu et al., 2022: PMID: 35600483). Furthermore, it is important to consider the timing and context of these changes. In *tak1* mutants, heart rate is not significantly altered at 3 dpf, the stage when trabeculation is first initiated. Again, the increase in heart rate observed at 5 dpf likely reflects a secondary or compensatory response to earlier cardiac dysfunction, rather than a primary driver of altered hemodynamics. We have now discussed this in the revised manuscript.

5. The analysis of trabeculation in tak1 and tab2 mutants is not sufficient. In Fig 1F, the sagittal cross sections for trabeculation measurement between control and mutant embryos are equivalent. It is advisable to use different representative images. Images should be frontal view of the hearts and clearly show the trabeculation in WT hearts and the lack of it in mutant hearts. In Fig 1G, instead of saying 'severely reduced trabeculation' in the graph, it might be better to quantify number of trabecular ridges. Similar comments are applicable to the tab2 mutant analysis in the supplement.

We thank the reviewer for this helpful suggestion. In our study, all control and mutant embryos were imaged from the same angle and mounted identically to ensure consistency. The zebrafish larva is oriented at approximately a 45-degree tilt, with the anterior end angled downward and the ventral side partially facing upward. This positioning allows both eyes to remain visible in the field of view providing a consistent anatomical reference for imaging. The images shown in Fig 1F are selected sections from whole-mount image stacks and reflect equivalent anatomical planes. However, due to the severe cardiac looping defects observed in *tak1* and *tab2* mutants by 6 dpf (developmental stage when trabeculation becomes more prominent), the heart morphology is markedly altered. Consequently, even though control and mutant embryos were imaged from the exact same angle and mounted identically, the resulting views appear different. This appearance is consistent across all mutant larvae. We have now included an alternative representative image that more closely aligns with the control orientation. Importantly, the relative positioning of cardiac chambers continues to highlight the morphological distortion caused by impaired looping.

We agree with the reviewer that presenting the trabeculation data as the number of trabeculae is more appropriate. Accordingly, we have now included this measure for both *tak1* (Fig 1G) and *tab2* (Fig S3D) mutants.

6. The analysis of the valve defects is not adequate for the tak1 and tab2 mutants. The valve defects are not equivalent in the mutants, as in tab2 have valve defects. Additionally, better images of the valves in the mutants need to be presented. This could be done with an appropriate transgenic line or the use of Alcama immunohistochemistry.

We thank the reviewer for this suggestion. Quantitative assessment of the heart valve defect was only feasible through cell number quantification, which is the approach we employed. For visualization, we initially attempted to mark the cardiac valves using the *Tg(kdrl:mCherry)* transgenic background; however, the fluorescent signal was relatively insufficient for high-resolution imaging. We therefore used Sox9 immunohistochemistry, which provided clear and distinct visualization of the valve structures. We have now performed Alcama/Sox9 immunostaining for both *tak1* and *tab2* mutants, as suggested, and included it in Fig S2G and S3H.

We were unable to identify any established methods in the literature for quantitatively assessing valve morphology at these early developmental stages. However, in an effort to provide an additional quantitative parameter, we measured the valve-to-atrium angle. While we observed a reduction in *tak1* mutants, this change likely reflects an overall defect in cardiac looping rather than a valve-specific abnormality. For this reason, we did not include this analysis in the manuscript.

7. It is not clear how the hearts would have increased proliferation of both atrial and ventricular cardiomyocytes, yet only a modest increase in atrial cardiomyocyte number. In addition, it is not clear how the authors are designating atrial and ventricular cardiomyocytes without appropriate markers.

We thank the reviewer for raising this point. It is possible that proliferative signals are activated in both chambers, but the survival, or differentiation of proliferating cells differs between the atrium and ventricle. Additionally, we observed considerable variation in the number of proliferating cells among the *Tak1* mutants´ventricles, which could contribute to the heterogeneity in proliferation outcomes. We have now addressed this point in the corresponding result section.

A straight line at the narrowest point of the atrioventricular canal was used as a defining anatomical landmark, facilitating clear distinction between the atrium and the ventricle. The atrial marker we had, anti-Myh6 (s46), and the Pcna antibody are both raised in mouse, which limits the simultaneous use of these markers in co-staining experiments. We have now put a note on this in the corresponding methods section.

8. The images of Mef2a/c should be presented with separate channels. Arrows and additional markers are needed to indicate the cells of interest for the reader. What cells are proliferating in Fig. 1h?

We have now added separate channels in Fig 1H and marked examples of proliferating cells.

9. Why were effects on gene expression from RNA-seq confirmed at different stages 3 dpf for tak1 mutants ant 5 dpf for tab2 mutants? Additionally, it is not clear that the transcriptomic changes observed do not simply reflected delayed differentiation of the embryos. This needs to be clarified.

We thank the reviewer for this comment and understand the potential confusion. The manuscript already includes the following explanation for stage selection: “For RT-qPCR, embryos at 3 dpf and 5 dpf for *tak1* and *tab2* mutants, respectively, were used, as these were the stages when mutants were fully penetrant and phenotypically distinguishable from their heterozygous and wild-type siblings from the same breeding.”

We specifically selected these phenotypically fully penetrant stages so that a higher proportion of homozygous mutant embryos could be reliably distinguished from heterozygous and wild-type siblings in crosses of heterozygous parents. This was important because we used approximately 100 embryos for cardiac extraction and a large number of clearly phenotypic embryos (i.e., homozygous mutants) was required for the experiments.

We understand the reviewer’s concern regarding potential developmental delay in the embryos. However, all experimental groups were stage-matched, and we did not observe any indication of delayed development. We have now added a note on this point in the manuscript.

10. Does treatment of zebrafish embryos with the Tak1 inhibitors result in similar defects as the mutants (increased atrial cardiomyocytes, decreased cardiac function, loss of trabeculation)?

We thank the reviewer for this valuable suggestion. We have now used two distinct pharmacological inhibitors of Tak1 (Takinib and 5ZO) and assessed cardiac morphology in terms of chamber size. Compared to takinib treatment, 5ZO-treated zebrafish display a more uniform and consistent phenotype (observed in 100% of larvae) that very closely recapitulates that of *tak1* mutants. In addition, we evaluated trabeculation in 5ZO-treated zebrafish and the resulting phenotypes were consistent with those observed in *tak1* mutants, thereby strengthening the evidence for Tak1 function in these processes. These data are included in Fig S5.

11. In Fig S3C, the representative atrium for the control group seems to be imaged at a distorted angle making appear smaller than normal. Thus, the atrial size difference cannot be justified on the basis of this Fig. It is advisable to change the representative image for control.

We thank the reviewer for pointing this out. As noted in our response to point 5, all control and mutant embryos were imaged under exact identical conditions, including the same angle and mounting orientation, to ensure consistency. At 5 dpf, due to further cardiac development and looping, the relative positions of the heart chambers differ compared to 3 dpf (see, for 5 dpf example, PMID: 35098309). Fig S3C shows a confocal microscopy image; however, cardiac chamber quantification was performed using stereo microscope images, with analysis focusing separately on the two chambers. We now included another representative image for the control larvae in Fig S3C where the atrium is not much obscured.

12. In Fig 3a, what the sections represent with respect to the heart is not clear. A schematic of where the sections were take could be used or image at a higher magnification. Importantly, it's not clear what are have marked as cardiomyocytes are actually cardiomyocytes and not another cell type, such as endocardial cells. Additional cardiomyocyte markers to show that expression should be used. A loss of trabeculation may be more consistent with defects in endocardial cilia and not myocardial cilia. It is also not clear why the image in the control does not have the Tak1 expression.

We thank the reviewer for this comment. Cilia staining in zebrafish cardiac tissue has proven technically very challenging. We have repeated the experiment multiple times, optimizing several parameters and testing different ciliary antibodies. To this end, we have repeated the experiment and now include DIC images to better orient the reader to the region of interest. Importantly, we validate the localization of Tak1 to the ciliary base area in cardiomyocytes. And we consistently observe loss of this staining in *tak1* mutants. Unfortunately, despite testing multiple markers, we were unable to include additional myocardial markers in zebrafish tissue sections (where primary cilia can be stained) due to conflicts between antibody host species and technical difficulties.

13. The levels of JNK1/2 were assessed following TAK1 inhibitor treatment. Are they affected in the TAK1 and TAB2 mutant cell lines or zebrafish.

We thank the reviewer for this point. We have now performed western blot analyses using whole-larvae lysates from 3 dpf zebrafish comparing *tak1^-/-^* and *tak1^+/+^* fish to assess the levels of phosphorylated JNK1a/b (pJNK1a/b) and total JNK1a/b. Here we show that the mutant larvae display reduced levels of JNK1a/b phosphorylation compared to wild-type controls (Fig 5N, 5O).

14. There seems to be a disconnect with the effects in zebrafish mutants, where it is not clear that cardiomyocyte differentiation is affected, and the cell culture model, in which TAK1 and TAB2 inhibition affect cardiomyocyte differentiation. This should be discussed.

We thank the reviewer for this insightful comment. We agree that the phenotypic differences between TAK1 loss in P19 cell cardiogenesis and zebrafish mutants should be discussed. One possible explanation for this discrepancy is that the *in vivo* environment provides regulatory features absent *in vitro*, including cellular heterogeneity, tissue-level signaling interactions, and compensatory genetic or signaling mechanisms. These factors may buffer early differentiation defects, such that the predominant phenotypes observed in zebrafish primarily reflect later aspects of cardiac morphogenesis, such as myocardial growth, ECM remodeling, or chamber morphology. We have now included a discussion of these points in the revised manuscript.

15. The coverage of the patient mutation data is lacking. What are the specific mutations that were mapped to TAK1 and are they predicted to be disease causing? This aspect of the manuscript should be better developed.

We thank the reviewer for this point. We have now added a supplementary Table (Table S1) with information about the type of variants identified in cases and controls. In addition, we have calculated the CADD score of each variant and used CADD > 25 as a measure of potentially disease-causing variants. The burden of variants with CADD > 25 was calculated to 3.3 and 11.5 for TAK1 and TAB2, respectively, thus supporting an association between sCHD and rare pathogenic variants in TAK1 and TAB2. Additional text has been added to the result section, and additional data is presented in Table S1 and Fig S1A-C.

Minor issues:

1. Fig S3E,F - Though tak1 operates downstream of tab2, tak1 mutants have no AV valve defects while tab2 mutants do. Does this imply that a different signaling effector downstream of tab2 is responsible for AV valve development.

We thank the reviewer for this insightful comment. While Tak1 is a well-established downstream effector of Tab2, the absence of AV valve defects, at least in terms of cell number, in *tak1* mutants compared to the clear phenotype observed in *tab2* mutants (an observation consistent with patient phenotypes) might indeed suggest that Tab2 likely regulates AV valve development through additional signaling pathways or effectors beyond Tak1. Tab2 is known to engage multiple downstream signaling cascades, and it is therefore plausible that other effectors contribute to AV valve formation. We have now incorporated this point into the Discussion of the manuscript.

2. There is a concern that some aspects of the defects in the zebrafish mutants are due to developmental delay and/or are secondary. It would helpful to provide better characterization of the onset of defects in the mutants.

We thank the reviewer for raising this important point. Developmental delay and secondary effects are important considerations when interpreting phenotypes in zebrafish. In our study, we carefully stage matched all experimental groups to ensure that control and mutant embryos were comparable at the time of analysis. We also aimed to comprehensively characterize the mutant phenotypes by assessing multiple parameters, in line with established approaches in the field. This allowed us to evaluate the consistency of the observed defects across different readouts. That said, we acknowledge that some aspects of the phenotype could arise as secondary consequences of earlier defects. We now added a note highlighting this point.

3. None of the graphs in any of the Figs have labels to indicate units.

We thank the reviewer for pointing this out. We have now added units to all graphs where applicable.

4. Figs should have stages directly indicated on the panels.

We have now indicated the developmental stages directly on the figure panels, in addition to including them in the figure legends for clarity.

5. Why is heart rate normalized in Fig. 1g? That is not standard.

The literature includes examples of presenting heart rate data both as raw values and as normalized values. Normalization was applied to account for external factors, such as room temperature and batch-to-batch variability, that can influence measurements. This approach allows for more reliable comparisons across experimental groups. Importantly, the overall results are consistent when using the raw data, which we now included instead of normalized data.

6. Labels in Figs should indicate what is being stained not the name of the antibody, for instance Almaca not ZN-8 in Fig. 1f, and the name of the transgene, for instance myl7:dsRed, so that the cells labelled are made clear in the Fig.

We thank the reviewer for raising this point. We now changed the labels accordingly.

7. The transgenic indications for zebrafish do not follow standard nomenclature rules.

We thank the reviewer for noticing this mistake. We have now corrected the transgenic zebrafish line mentions that did not follow standard nomenclature rules.

8. The lower panel in Fig. 1h is mislabeled.

We thank the reviewer for noting this mistake. We now corrected the label.

**Reviewer 2:**

In this manuscript, the potential function of Transforming Growth Factor-Beta-Activated Kinase 1 (TAK1), a player in the non-canonical arm of TGFbeta/BMP signalling, is explored. Mutations in TAK1 and genes encoding associated proteins TAB2 (TAK1 binding protein) and PRKACA (which phosphorylates TAK1) have been described in patients with congenital heart disease (CHD). However, how perturbation of this pathway leads to heart and extracardiac defects is largely unknown.

In analyzing exomes from roughly 3,800 syndromic and non-syndromic CHD patients, an increased burden of damaging variants in TAK1 and TAB2 were observed (vs 48,000 controls). Tak1 and tab2 zebrafish mutants are generated, with both displaying cardiac abnormalities including a lack of trabeculation at 5 days post-fertilization, increased atrial size, and dysregulated cardiac gene expression at 3 days of development (tak1 mutants). Craniofacial defects are also observed, consistent with human syndromes associated with TAK1 and TAB2. The authors go on to convincingly show that Tak1 localizes to the base of the primary cilium in multiple contexts - embryonic zebrafish heart, iPSCs cardiac organoids, RPE-1 cells and differentiating P19 cells, with human CHD-associated mutations (2) affecting ciliary localization. Phosphorylation of Tak1 at the base of the cilia is seen during cardiac differentiation (P19 model) and following TGFb1/BMP2 stimulation, with Tak1 loss or inhibition
affecting cardiomyogenesis of P19 cells.

Overall this is a well-written manuscript (some typos are evident) that convincingly shows a role for TAK1 in heart development, likely through localization to the base of the primary cilium. A potential role for TAK1 as an integrator/modifier of multiple signaling pathways during cardiac development is an interesting concept that certainly merits further study.

Major Comments:

1. The statement that "TAK1 coordinates a significant part of gene-networks involved in development of the myocardium, cardiac ECM and cardiac smooth muscle cells", based on the RNA-seq analysis of 3dpf hearts, seems like a stretch to me. Mutant hearts with phenotypic defects are analyzed and several cardiac GO terms are indicated in down-regulated genes. However this is in hearts that are already likely experiencing defective flow, contractility and other key factors that affect cardiac development and gene expression. many of the genes listed (hand2, etc.) are key players in cardiac development whose mutation alone causes far more severe cardiac defects.

We thank the reviewer for raising this important point. We agree that one should be cautious when interpreting transcriptomic changes in hearts that are already exhibiting defects. Our intention was not to claim that Tak1 is the sole driver of myocardial, ECM, or smooth muscle development, but rather that it appears to coordinate a subset of gene networks relevant to these processes, as indicated by the enrichment of cardiac-related GO terms among the downregulated genes. We acknowledge that some genes, such as *hand2*, are well-established critical regulators whose mutation produces more severe phenotypes. The observed changes in our RNA-seq data likely reflect a combination of direct Tak1-dependent transcriptional effects and secondary responses to altered flow, contractility, or other developmental perturbations. We have revised the text to clarify this in the manuscript.

2. Some commentary should be made on the temporal dynamics of Tak1 phosphorylation (Fig 3), where maximal signal is achieved 30-90 minutes after acute TGFb1 or BMP2 stimulation. Is this in line with other TGFbeta-stimulated signaling events, or what has been shown for TAK1 previously?

In RPE-1 cells, used here as a model for ciliary signaling, we evaluated TAK1 activation over a time course consistent with prior studies of TGFB superfamily signaling (PMID: 23746451, 29514088, 37706312). This approach also aligns with other cell culture studies examining TAK1 phosphorylation by western blot analysis (PMID: 24963009, 37706312). We acknowledge that TGFB/BMP signaling can be evaluated across a broad range of timepoints, but the intervals selected in our studies concretely corroborate with our previous efforts to investigate the axis of ciliary TGFB/BMP/TAK1 signaling (PMID: 23746451, 29514088). Importantly, this also allowed us to investigate potential crosstalk between TAK1 and R-SMAD signaling, as pointed out by reviewer 4, comment 3, for which we have now included new data to show that TAK1 acts upstream of SMAD2/3 signaling (Fig S10).

3. It is difficult to reconcile the later effects on heart development seen in tak1 zebrafish mutants with the striking effects seen in P19 cardiogenesis, where the initial events of cardiomyocyte differentiation are strongly affected. The authors should attempt to address this in the Discussion, and at least speculate on why this may be.

We thank the reviewer for this insightful comment. We agree that the phenotypic differences between TAK1 loss in P19 cell cardiogenesis and zebrafish mutants should be discussed. Please see response to reviewer 1, comment 14.

4. To help with point 3, have the authors tried using the TAK1 inhibitors employed for P19 studies during different stages of zebrafish cardiac development? Is it clear the zebrafish do not have other TAK1 family members?

We thank the reviewer for this suggestion. We have now performed pharmacological inhibition experiments using two independent Tak1 inhibitors (Takinib, 5ZO), with treatment initiated at 24 hpf. Notably, inhibition of Tak1 produced cardiac phenotypes closely comparable to those observed in the *tak1* mutant zebrafish (Fig S5), providing further support for the involvement of Tak1 in this process.

Compared to Takinib treatment, 5ZO-treated zebrafish displayed a more uniform and consistent phenotype (observed in 100% of larvae) that very closely recapitulated that of *tak1* mutants. We therefore used 5ZO to assess the effects of earlier treatment. A subset of embryos (30%) treated with 5 uM 5ZO prior to gastrulation (30% epiboly) showed pronounced morphological abnormalities, including reduced axial elongation, when assessed at 24 hpf. By 72 hpf these embryos exhibited phenotypes similar to those observed in embryos treated at 24 hpf. To minimize potential interference with early embryonic patterning during gastrulation and segmentation stages, subsequent experiments were conducted by administering 5ZO at 24 hpf.

Minor Comments:

1. Some description of the zebrafish CRISPR mutants generated should be provided: Fig S1 shows the sequence of indels, but what portion of the gene and associated protein structures are affected? Are these alleles likely to undergo nonsense-mediated decay and resulting transcriptional adaptation?

We thank the reviewer for this comment. We have now included a schematic of the predicted protein sequences resulting from the CRISPR-induced mutations, showing which portions of the protein are affected (Fig S1F). Consistent with this, we observe a significant reduction in *tak1* mRNA levels (Fig S6B) and a complete loss of detectable protein (Fig S1G). While any transcriptional adaptation may occur, it appears to be insufficient to fully compensate, as evidenced by the numerous phenotypes observed in these mutants. Our inhibitor data further support the observed phenotypic outcomes.

2. It is hard to see how the cardiac defects observed in tak1 and tab2 morphants phenocopy mutants, given the heart images provided in Fig S4. Further, tab2 morphants appear far more severely affected morphologically (Fig S4b).

We thank the reviewer for this comment. We acknowledge concerns regarding morpholino specificity and have therefore removed the morphant data from the revised manuscript.

We have now performed pharmacological inhibition experiments using two independent inhibitors, Takinib and 5ZO, both of which produced cardiac phenotypes comparable to those observed in *tak1* mutants, thereby strengthening the evidence for Tak1 function in these processes.

3. The significance of the Meckel's-palatoquadrate angle with respect to craniofacial development should be explained. Does this (and eye spacing) reflect a larger head?

We thank the reviewer for this comment. The Meckel’s-palatoquadrate (M-PQ) angle is commonly used as a quantitative measure of craniofacial development, as it reflects the relative positioning and growth of jaw elements. A wider Meckel’s-palatoquadrate angle may give the appearance of a broader or differently shaped face, but they do not necessarily indicate an overall increase in head size, for which we did not observe any substantial changes. Similarly, eye spacing provides a measure of craniofacial patterning and positioning. We have clarified this point in the revised manuscript to emphasize that these metrics specifically reflect craniofacial morphology.

4. In describing the results for Fig 3E/F, it should be made clear in the text that these are also RPE-1 cells being used (as in the previous paragraph).

We thank the reviewer for pointing this out. We have clarified in the text that the experiments shown in Fig 3E and 3F were also performed using RPE-1 cells.

**Reviewer 3:**

In this manuscript, the authors investigate how mutations in human TAK1 and TAB2 contribute to early embryonic development defects, specifically craniofacial and cardiac congenital abnormalities. Using zebrafish as an in vivo model, they demonstrate that tak1 and tab2 are essential genes, with loss-of-function mutations leading to severe cardiac and craniofacial malformations. They further explore Tak1 protein localization and report its enrichment at the base of primary cilia in cardiac cells. This observation leads them to complement their in vivo studies with in vitro models, including gastruloids and P19 cells. Their data suggest that Tak1 localizes to primary cilia in response to BMP signaling, which is required for cardiomyocyte differentiation. Finally, the authors examine two human TAK1 variants identified in patients and show that these variants exhibit impaired localization to the primary cilium.

Overall, the manuscript is well written, and the data largely support the authors' conclusions. The Figs are clear and effectively illustrate the findings. However, while the authors demonstrate that tak1 and tab2 are required for embryonic, particularly cardiac, development, the conclusion that this occurs via BMP/TGF-β signaling at the cilium is primarily supported by in vitro evidence. Additional in vivo data are necessary to substantiate this claim.

Major Comments:

1. Tak1 Localization at the Primary Cilium In Vivo:

The claim that TAK1 localizes to the primary cilium is convincingly supported by the in vitro data. However, the in vivo evidence presented in Fig 3A is limited. The image shows many ciliated cells, but only a single example of clear Tak1 localization at the cilium base. This raises several important questions:
o What is the pattern of Tak1 localization in other ciliated cells? Is Tak1 present at the base of all cilia or only in a specific subset?

o Is this localization specific to cardiomyocytes? The authors have not used a cardiomyocyte-specific marker to support this claim. It would be important to determine whether TAK1 is also present in other cell types, such as endocardial or endothelial cells.

We thank the reviewer for this comment. We have now presented new images, where the Tak1 localization to the cilium in cardiomyocytes is more recognizable (see also response to reviewer 1, comment 12).

2. Non-Canonical BMP Signaling via Tak1/Tab2 In Vivo:

The title and abstract state that non-canonical BMP signaling functions through Tak1/Tab2. While the authors provide supporting in vitro data, there is insufficient evidence for this mechanism in vivo, especially in relation to the cardiac defects observed in tak1 and tab2 mutants. The authors should consider performing in vivo experiments to assess:

o Whether BMP activation leads to Tak1 relocalization to the cilium.

o Whether manipulation of BMP signaling affects cardiac development in a Tak1/Tab2-dependent manner.

We consider this an important point, but also one that we have found technical limitations in our abilities to address. The extracellular receptor domain of both the BMP and TGFB receptor is different in fish, which makes our cellular assays incompatible with zebrafish (PMID: 12697700, 9834197). Meanwhile, manipulating BMP or TGFB itself leads to cardiac developmental defects (PMID: 19232521, 35522174), resulting in difficulties deciphering the concrete contributions of Tak1/Tab2 within this system, unless these are specifically interrogated. We now provide evidence that treating zebrafish with the TAK1 inhibitors we use in our *in vitro* experiments results in zebrafish that phenocopy *tak1* mutants.

While we acknowledge that we cannot directly assess BMP-dependent Tak1 relocalization to the cilium *in vivo* at this stage, our combined *in vitro* and *in vivo* data support a functional role for Tak1/Tab2 downstream of BMP signaling.

Additionally, the relationship between BMP signaling and the observed cardiac phenotypes remains unclear. Previous studies suggest that reduced BMP signaling leads to a decrease in cardiomyocyte number, while the tak1 mutants described here show an increase in atrial cells. These apparent discrepancies must be addressed to clarify the proposed role of Tak1 in non-canonical BMP signaling during heart development.

We thank the reviewer for this important point. We agree that the relationship between BMP signaling and the observed cardiac phenotypes requires clarification. Previous studies demonstrating reduced cardiomyocyte number upon decreased BMP signaling primarily describe effects on specific cardiac regions, particularly the inflow tract (IFT), rather than a global reduction in all cardiomyocytes (PMID: 41480663). This indicates that BMP signaling has spatially distinct roles during heart development. We have now added a note that the precise relationship between BMP/TGFB/TAK1 signaling and the observed cardiac phenotypes remains to be further elucidated.

Minor Comments:

1. Extracellular Matrix (ECM) Defects:

The authors report changes in ECM-related genes in the transcriptomic data from tak1 and tab2 mutants. To support these claims, the authors should perform ECM-specific staining to determine whether structural changes are evident in the mutant hearts.

We thank the reviewer for this insightful suggestion. We agree that further investigation of ECM composition and structure would be highly informative. However, as multiple ECM components are affected, a single staining approach would likely provide only a limited view of these alterations. We therefore believe that a comprehensive analysis of ECM remodeling would be better suited for a dedicated follow up study. To address this point, we have now included a statement in the manuscript acknowledging that further work will be required to fully assess ECM-related changes in these mutants.

2. Morpholino Versus Mutant Phenotypes:

The phenotypes observed following morpholino (MO) knockdown appear more severe than those in genetic mutants. For instance, heterotaxy is observed in MO-injected embryos but not described in mutants. The MO-treated embryos also exhibit nonspecific defects, such as reduced head and eye size, which are absent in mutants. These differences suggest potential off-target effects, and caution is warranted in interpreting the MO results. It may be advisable to omit the morpholino data from the manuscript unless further validated.

We thank the reviewer for this comment. We acknowledge concerns regarding morpholino specificity and have therefore removed the morphant data from the revised manuscript.

We have now performed pharmacological inhibition experiments using two independent Tak1 inhibitors, Takinib and 5ZO, both of which produced cardiac phenotypes closely comparable to those observed in *tak1* mutants (Fig S5).

**Reviewer 4:**

This manuscript presents a compelling and well-structured study examining the role of TAK1 (MAP3K7) in heart development, with a novel focus on its localization and function at the primary cilium during cardiogenesis. By combining human genetic data, zebrafish models, mammalian cell systems (RPE-1, P19CL6), and gastruloids, the authors provide strong evidence that TAK1, along with its upstream regulators TAB2 and PKA-Ca, acts as a non-canonical TGFB/BMP signaling hub at the ciliary base. The study is mechanistically insightful, and the manuscript is generally clear and logically organized. However, there are several issues regarding data presentation, clarity, and mechanistic interpretation that should be addressed.

Specific Comments:

1. The authors show TAK1 localization to the ciliary base in cardiomyocytes. Is this localization heart-specific, or is TAK1 similarly localized in other tissues? If its expression and localization are broader, it would be important to consider whether TAK1's role at the cilium is more general, beyond cardiomyogenesis. Additional analyses or discussion on its function in other organs and tissues would strengthen the manuscript.

We thank the reviewer for this point, and indeed TAK1 localizes to primary cilia outside the cardiac tissues, as also shown for RPE-1 cells. We have now, as suggested by the reviewer, included a remark on this in the main text implying a broader role for TAK1 signaling during development in line with the developmental defects observed in TAK1 patient variants.

2. The manuscript refers to TAK1 localization at the cilium base, but it is unclear whether this refers to the basal body, transition zone, or centrosome. Please clarify this point and include co-staining with specific markers such as gamma-tubulin or IFT88 to confirm subcellular localization.

We thank the reviewer for this suggestion and agree that it is valuable to show the sub-ciliary localization of TAK1. We have now performed additional co-staining experiments to more precisely define its subciliary localization. To this end, TAK1 was co-stained with a ciliary marker alongside ciliary base (Centrin-2), basal-body distal appendages (CEP164) and IFT88, which marks the ciliary base and can be observed along the length of the cilium (Fig S8).

3. Fig 5n depicts canonical SMAD signaling alongside non-canonical pathways. Do the authors have experimental evidence showing changes in SMAD-dependent signaling (e.g., pSMAD2/3 levels) in TAK1 mutant or inhibitor-treated models? Inclusion of such data would provide a more complete picture of how TAK1 intersects with canonical TGFB/BMP signaling.

We agree with the reviewer that the model shown in Fig 5N (now Fig 5P) could benefit from supporting experiments. We proposed the model based on data showing TAK1 promotes a crucial linker-region phosphorylation in SMAD2/3, that is necessary for receptor-mediated C-Terminal phosphorylation of SMAD2/3 (PMID: 23546880, 25849436). To validate these results, we have performed WB analyses on whole-cell lysates of both TGFB-1 and BMP2 stimulated RPE-1 cells in the absence or presence of 500 nM 5ZO. These experiments show, consistent with the model we proposed and the literature, that TAK1 inhibition ablates C-Terminal phosphorylation of SMAD2/3. We likewise show that TAK1 inhibition does not have any effect of the C-Terminal phosphorylation of SMAD1/5, suggesting that TAK1 intersects differentially between members of the TGFB superfamily signaling components.

4. TAK1 phosphorylation at the cilium is shown at discrete time points. Could the authors provide a more detailed temporal analysis? It would be helpful to clarify whether TAK1 activation is sustained or transient, and whether it differs between TGFB and BMP ligand stimulation.

We thank the reviewer for this suggestion. We have selected discrete time points for the stimulation assays, since this is customary in both the cilia and TGFB-signaling fields, as noted in our response to reviewer 2, comment 2. Within the chosen time points, TAK1 activation in response to TGFB1 and BMP2 stimulation appears to follow a similar transient pattern.

5. The zebrafish data indicate defects in trabeculation and chamber morphology. Is there evidence from marker expression or lineage tracing that specific cardiac cell types (e.g., endocardial, myocardial, epicardial) are preferentially affected? Are there any observed defects in the outflow tract?

We thank the reviewer for this important question. While we agree that lineage tracing would be a powerful approach to definitively determine whether specific cardiac cell types are preferentially affected, such analyses are beyond the scope of the current study and we do not currently have lineage tracing tools established in our lab.

To further assess the outflow tract (OFT), we performed fluorescent *in situ* hybridization for *elastin b* (*elnb*), a well-established OFT marker, and observed a reduction in the *elnb2*-positive area in both *tak1* and *tab2* mutant embryos. These results indicate that Tak1/Tab2 signaling is also involved in OFT development. We have now included this data as Fig S4.

6. The valve morphology in Fig. S2G appears abnormal. Please clarify these findings and consider additional staining or quantification. The same concern applies to the tab2 mutant valve phenotype shown in Fig. S3E.

We thank the reviewer for this suggestion. Quantitative assessment of the heart valve defect was only feasible through cell number quantification, which is the approach we employed. For visualization, we initially attempted to mark the cardiac valves using the *Tg(kdrl:mCherry)* transgenic background; however, the fluorescent signal was relatively insufficient for high-resolution imaging. We therefore used Sox9 immunohistochemistry, which provided clear and distinct visualization of the valve structures. We have now performed Alcama/Sox9 immunostaining for both *tak1* and *tab2* mutants, to more effectively visualize the valves.

We were unable to identify any established methods in the literature for quantitatively assessing valve morphology at these early developmental stages. However, in an effort to provide an additional quantitative parameter, we measured the valve-to-atrium angle. While we observed a reduction in *tak1* mutants, this change likely reflects an overall defect in cardiac looping rather than a valve-specific abnormality. For this reason, we did not include this analysis in the manuscript.

7. The ventricle in the tab2 mutant appears enlarged (Fig. S3C), and the atrium in the control looks morphologically abnormal when compared to Fig. 1F. It would be important to show dissected hearts, as in Fig. 1H, for consistency and clearer assessment.

We thank the reviewer for this comment. The top panel of Fig 1F shows a 3 dpf heart, whereas Fig S3C corresponds to 5 dpf, and the observed differences reflect normal developmental progression. We have now indicated the developmental stages directly on the figure panels, in addition to including them in the figure legends for clarity. Several *tab2* mutants display ventricular enlargement, as indicated by the individual data points in the ventricular area quantification.

8. The morpholino experiments are described as producing "similar" phenotypes, but no heart images or quantitative measurements are provided. Please include representative images and phenotypic quantification to support these claims.

We thank the reviewer for this comment. We acknowledge concerns regarding morpholino specificity and have therefore removed the morphant data from the revised manuscript.

We have now performed pharmacological inhibition experiments using two independent inhibitors, Takinib and 5ZO, both of which produced cardiac phenotypes closely comparable to those observed in tak1 mutants (Fig S5).

9. Fig. 1J . Please include lateral views to better visualize the craniofacial phenotype. Also, specify which fin was measured (e.g., pectoral fin). Are other fins, such as the tail fin, also affected?

We thank the reviewer for the suggestion. Lateral views have now been included in Fig 1J and S3J. The measurements were performed on the pectoral fin, as now specified in the figure legends. Additionally, a bright-field lateral image of *tak1* mutant has been included to further illustrate the phenotype (Fig S2B). No other obvious phenotypes were observed in the other fins. We have now noted it in the corresponding result section.

10. While gastruloids offer a 3D system to study early development, the rationale for choosing this model over more physiologically relevant in vivo systems (e.g., mouse embryos) is not clearly justified. Why were mechanistic studies conducted in cell lines rather than extending validation to the gastruloid model?

We thank the reviewer for raising this important question. We used gastruloids as a model for the earliest stages of cardiogenesis, as these stages are challenging to access and stain in embryos and would require animal sacrifice (PMID: 40472086). Previous studies in our laboratories identified a role for ciliary TGFB signaling in regulating heart field development (PMID: 29934521), and our zebrafish bulk RNAseq data revealed downregulation of multiple cardiac transcription factors. Based on this, we considered gastruloids to be a suitable system to interrogate early cardiac development in the context of ciliary TAK1 and its phosphorylation.

The feasibility of using TGFB superfamily ligands to stimulate TAK1 in gastruloids to interrogate the ciliary signaling module is questionable, given the role TGFB superfamily signaling itself has on cardiogenesis and tissue patterning. We therefore chose to use 2D cell culture systems to specifically evaluate the ciliary modulation of the pathway. This in turn allowed us to more readily use inhibitors, KO and transfections to evaluate how TAK1 operates at the primary cilium, and the consequences of its removal. Functionally, we attempted pharmacological inhibition of TAK1 in gastruloids using both Takinib and 5ZO. However, these experiments were inconclusive, as vehicle-treated gastruloids did not exhibit sufficient cardiogenesis to allow meaningful comparison. Nevertheless, our findings linking primary cilia to TAK1 signaling in gastruloids exemplifies how this model could be used for future studies in ciliary signaling in the earliest development periods.

11. It would be informative to indicate proliferating cardiomyocytes with arrows in Fig. 1H, particularly to support conclusions about proliferation defects.

We thank the reviewer for pointing this out. We have now added separate channels in Fig 1H and marked examples of proliferating cells.

12. In Fig. 3B (lower panels), the localization of TAK1 and ARL13B is unclear: are the signals overlapping or simply adjacent? The green arrowhead also seems mispositioned. The same issue occurs in Fig. 4F. It is confusing when both co-localization and parallel arrangements are shown within the same Fig panels. Please clarify.

We agree that the clarity of the figure panels is critical for proper interpretation and appreciate the reviewer highlighting this point. The localization patterns of TAK1 and ARL13B are overlapping, and when they are adjacent it is because the panel shows a shifted image, which aims to show specifically TAK1 localization throughout the entire cilium. This is also recommended to aid color blind people in viewing the contents of the figure. We have now annotated and described the figures more clearly to prevent any future confusion regarding these panels.

13. The tab2 mutant data shown in Fig. S3 are central to the overall narrative and should be moved to the main Figs. Similarly, the informative signaling data in Figs. S5A, B would be more impactful if moved to Fig 2.

We thank the reviewer for the suggestion. Figs S5A, B (now S6A, B) provide supporting data for Fig 2, thus we believe they are best presented as part of the supplementary material.

Regarding the *tab2* mutant data in Fig S3, if the editor prefers, we would be happy to move this figure to the main figures.
