# Supplementary material for: TAK1 operates at the primary cilium in non-canonical TGFB/BMP signaling to control heart development
Source: PLoS Biol. 2026 Aug 4;24(8):e3003902. doi: 10.1371/journal.pbio.3003902 (PMC13436766; doi:10.1371/journal.pbio.3003902)
Supplement: Supplementary file 5 [file pbio.3003902.s021.docx]

We sincerely thank the reviewers for their continued careful evaluation of our manuscript and for their valuable additional comments and suggestions.

**Reviewer 1:**

In the revised manuscript from Doganli et al, the authors explore the requirement of TAK1 and TAB2, and other ciliary components in heart development. Human exome sequencing data implicated TAK1 and TAB2 as a risk factor in syndromic congenital heart defects. To model mutations in TAK1 and TAB2 in vivo, the authors generated zebrafish mutants for tak1 and tab2, which exhibited cardiac trabeculation defects, as well as craniofacial and limb defects, potentially reflecting FMD and CSCF syndromes found with human mutations. Pharmacological inhibition of Tak1 generated similar defects as the tak1 mutants. Bulk RNA sequencing of tak1 mutant hearts showed that loss of tak1 had a decrease in expression of transcription factors that promote heart development, sarcomeric proteins, and cardiac ECM genes. The authors utilized in vitro cell culture systems to show that TAK1, TAB2, and PKC-Cα localize to the primary cilium of differentiating cardiomyocytes. Human mutant versions of TAK1 do not localize to the base of cilia. Inhibition of TAK1 and TAB2 via CRISPR/Cas9 generated mutant lines and pharmacological inhibition of TAK1 blocked cardiomyocyte differentiation in vitro. TGFB addition is able to stimulate TAK1 phosphorylation and JNK1/2 phosphorylation at the base of cilia is inhibited via pharmacological inhibition of TAK1 in vitro. The authors conclude that their research provides insights into the regulation of TAK1 within the cilia and sheds new light on its role in cardiomyocyte development and potentially the causes of syndromic congenital heart defects.

The revised manuscript is much improved over the previous submission. It is appreciated that the authors we responsive to the majority of the previous critiques. The interpretations are more in line with their presented data. The actual mechanism underlying the trabeculation and valve defects in the zebrafish is not explained nor are the differences between the in vitro and in vivo data. However, the authors address the latter issue in the discussion. Overall, there is a significant amount of data supporting the molecular mechanism that is proposed in this improved manuscript, which may support a novel mechanisms of action within the cilia underlying syndromic congenital heart defects.

The statistical analysis used appears appropriate. Comments regarding supplementary data are below. Accession numbers for data have been supplied.

I have a some remaining minor suggestions that the authors may want to consider with respect to their final manuscript.

Minor issues:

1. On page 12, the sentence with "displayed no defect" is awkward and should be edited.

We thank the reviewer for pointing this out. We have now revised the sentence.

2. The increased heart rate in the mutants seems statistically different, but is this biologically meaningful? The mean for wt and tak1 mutants is 140 bpm vs 150 bpm. It seems to receive greater emphasis than needed in the manuscript. What this means is nor is its relationship to trabeculation clear and the cause of the tachycardia, like effects of Tak1 on pacemaker cells, are not examined.

We thank the reviewer for this comment. We have now toned it down in the discussion.

3. Some Figures are presented out of order, in particular Fig. S4A.

We thank the reviewer for pointing this out. We have now moved the text accordingly

4. Fig. S4A is difficult to interpret due to the angles used for imaging. It is hard to see the bulbus is actually reduced in size in the mutants. Presenting the different imagining channels may make this point clear.

We thank the reviewer for this point. We have now added different channels to the figure.

5. The "normalized atrial area" as in Fig. S5A and S5B is still not a good way to examine chamber size compared to the quantification the authors have not performed. It could have a lot of different interpretations that are not really covered. This analysis should be qualified within the text.

We thank the reviewer for this comment. We have now revised the Methods section to clarify that the chamber dimensions were quantified as two-dimensional areas from single-plane fluorescent images and that this approach provides an indirect estimate of chamber size rather than a direct measurement of chamber volume.

6. On page 16, the "data not shown" might be good to show in the supplemental data, as the staining in the Fig. 3A is relatively minimal.

We have now included the data in Figure S7A. Similarly, we have included equivalent stainings of primary cilia outside clusters of cardiomyocytes in gastruloids (Figure S7E), which align with our observations in the developing heart of zebrafish larvae.

7. In Fig. 4F, the insets of Ac-tub/DAPI/TAB2 are pixelated.

We have updated the panel such that it is not pixelated anymore in the uploaded TIFF file.

8. It might be good to show to examine pSmad2/3 in the tak1 and tab2 mutant hearts, like they did with the RPE-1 cells.

We agree that assessing pSmad2/3 levels in *tak1* and *tab2* mutant hearts would be informative. We have attempted to perform Western blot analysis for pSmad2/3 in zebrafish (whole embryo lysates, due to the limited amount of cardiac tissue) in this context as well as in related ongoing projects. However, we were unable to obtain reliable and reproducible results.

9. With regard to interpretations of the RNA-seq data, the associated with terms related to fibroblasts likely reflects changes in epicardial cells, which is 3rd on the list in Fig. 2D, or the bulbus. The zebrafish heart does not have fibroblasts at that stage.

We thank the reviewer for this important comment. We agree that the annotation of “fibroblasts” requires careful interpretation in the context of the developing zebrafish heart. The enrichment analysis was performed using gene signatures derived from human embryonic hearts, and therefore reflects transcriptional similarity to mammalian cardiac cell-type programs rather than a direct one-to-one mapping of zebrafish cell types. We have revised the text to clarify this limitation.

**Reviewer 3:**

The authors have adressed most of my concerns but not all.

- Based on the images in Fig.3A and without a cardiomyocyte specific marker, they cannot conclude that Tak1 localizes to primary cilia in cardiomyocytes in zebrafish. The cilium can be seen inside the heart but whether the cilium is on an endocardial or myocardial cell in not evident. Therefore, they should adjust their conclusion that TAK1 localizes to cilia in cardiomyocytes in zebrafish.

We thank the reviewer for this important comment. We have revised the Figure 3A and the text accrodingly to better reflect Tak1 localization.

- In addition, the TAK1 localisation in gastruloids also indicates that it is not exclusive to nkx2.5+ cells but also present in other (nkx2.5-) cells. Evidence that TAK1 operates in cardiomyocytes is very weak and cardiac phenotypes observed can also be explained by effects on other (non-myocardial) cells. In addition, TAK1 has very different effects on cardiomyocytes in vitro versus in vivo experiments. Therefore they need to tune down the suggestion that TAK1 has a specific role in cardiomyocytes and cardiomyogenesis and rather refer to a role of TAK1 in cardiac development.

We thank the reviewer for this comment. We agree that TAK1 is not restricted to nkx2.5+ cells. We have now added further information on ciliary localization of TAK1 outside the cardiomyocyte clusters, supporting the findings from zebrafish larvae, indicating that ciliary Tak1 functions beyond the heart and offering a mechanistic link to the extracardiac phenotypes associated with TAK1 variants in FMD, CSCFS and THS.

- Methods section: Please include the temperature at which cardiac function was assesed as heart rate and cardiac output are influenced by temperture.

We thank the reviewer for this important comment. We have now clarified in the Methods section that zebrafish larvae were removed from the incubator and allowed to acclimate to room temperature (approximately 22°C) for 10 minutes prior to cardiac function assessment.

**Reviewer 4:**

The authors have addressed my major concerns. I do not have any further comments.

We thank the reviewer for the positive evaluation and are pleased that our revisions have addressed the concerns raised.
