# Supplementary material for: TAK1 operates at the primary cilium in non-canonical TGFB/BMP signaling to control heart development
Source: PLoS Biol. 2026 Aug 4;24(8):e3003902. doi: 10.1371/journal.pbio.3003902 (PMC13436766; doi:10.1371/journal.pbio.3003902)
Supplement: Peer review history

# Author comment: Author response to Decision Letter 0

**DOI:** 10.1371/journal.pbio.3003902.r001

1 May 2026

Attachment Submitted filename: Response to PlosBiology-28Apr2026.docx

# Editor report: Decision Letter 0

**DOI:** 10.1371/journal.pbio.3003902.r002

**Authors:** Ines Alvarez-Garcia

12 May 2026

Dear Dr Larsen,

Thank you for submitting the revision of your manuscript entitled "TAK1 operates at the primary cilium in non-canonical TGFB/BMP signaling to control heart development" for consideration as a Research Article by PLOS Biology.

We have had a look at the revision and we would like to send it back to the original reviewers to have another look. However, as his is a new submission, before we can send your manuscript to reviewers, we need you to complete your submission by providing the metadata that is required for full assessment. To this end, please login to Editorial Manager where you will find the paper in the 'Submissions Needing Revisions' folder on your homepage. Please click 'Revise Submission' from the Action Links and complete all additional questions in the submission questionnaire.

Once your full submission is complete, your paper will undergo a series of checks in preparation for peer review. After your manuscript has passed the checks it will be sent out for review. To provide the metadata for your submission, please Login to Editorial Manager (https://www.editorialmanager.com/pbiology) within two working days, i.e. by May 14 2026 11:59PM.

Feel free to email us at plosbiology@plos.org if you have any queries relating to your submission.

Kind regards,

Ines

--

Ines Alvarez-Garcia, PhD

Senior Editor

PLOS Biology

ialvarez-garcia@plos.org

# Author comment: Author response to Decision Letter 1

**DOI:** 10.1371/journal.pbio.3003902.r003

12 May 2026

Attachment Submitted filename: Response_to_PlosBiology-28Apr2026_auresp_1.docx

# Peer review: Decision Letter 1

**DOI:** 10.1371/journal.pbio.3003902.r004

**Authors:** Ines Alvarez-Garcia

12 Jun 2026

Dear Dr Larsen,

Thank you for your patience while we considered your revised manuscript entitled "TAK1 operates at the primary cilium in non-canonical TGFB/BMP signaling to control heart development" for publication as a Research Article at PLOS Biology. This revised version of your manuscript has been evaluated by the PLOS Biology editors, the Academic Editor and three of the original reviewers.

Based on the reviews (attached below), we are likely to accept this manuscript for publication, provided you satisfactorily address the remaining points raised by Reviewers 1 and 3. Please also make sure to address the data and other policy-related requests stated below my signature.

As you address these items, please take this last chance to review your reference list to ensure that it is complete and correct. If you have cited papers that have been retracted, please include the rationale for doing so in the manuscript text, or remove these references and replace them with relevant current references. Any changes to the reference list should be mentioned in the cover letter that accompanies your revised manuscript.

In addition to these revisions, you may need to complete some formatting changes, which you will receive in a follow up email. A member of our team will be in touch with a set of requests shortly. If you do not receive a separate email within a few days, please assume that checks have been completed, and no additional changes are required.

We expect to receive your revised manuscript within two weeks.

To submit your revision, please go to https://www.editorialmanager.com/pbiology/ and log in as an Author. Click the link labelled 'Submissions Needing Revision' to find your submission record. Your revised submission must include the following:

- a cover letter that should detail your responses to any editorial requests, if applicable, and whether changes have been made to the reference list

- a Response to Reviewers file that provides a detailed response to the reviewers' comments (if applicable, if not applicable please do not delete your existing 'Response to Reviewers' file.)

- a track-changes file indicating any changes that you have made to the manuscript.

NOTE: If Supporting Information files are included with your article, note that these are not copyedited and will be published as they are submitted. Please ensure that these files are legible and of high quality (at least 300 dpi) in an easily accessible file format. For this reason, please be aware that any references listed in an SI file will not be indexed. For more information, see our Supporting Information guidelines:

https://journals.plos.org/plosbiology/s/supporting-information

*Published Peer Review History*

Please note that you may have the opportunity to make the peer review history publicly available. The record will include editor decision letters (with reviews) and your responses to reviewer comments. If eligible, we will contact you to opt in or out. Please see here for more details:

https://plos.org/published-peer-review-history/

*Press*

Should you, your institution's press office or the journal office choose to press release your paper, please ensure you have opted out of Early Article Posting on the submission form. We ask that you notify us as soon as possible if you or your institution is planning to press release the article.

*Protocols deposition*

To enhance the reproducibility of your results, we recommend that if applicable you deposit your laboratory protocols in protocols.io, where a protocol can be assigned its own identifier (DOI) such that it can be cited independently in the future. Additionally, PLOS ONE offers an option for publishing peer-reviewed Lab Protocol articles, which describe protocols hosted on protocols.io. Read more information on sharing protocols at https://plos.org/protocols?utm_medium=editorial-email&utm_source=authorletters&utm_campaign=protocols

Please do not hesitate to contact me should you have any questions.

Sincerely,

Ines

--

Ines Alvarez-Garcia, PhD

Senior Editor

PLOS Biology

ialvarez-garcia@plos.org

------------------------------------------------------------------------

DATA POLICY: IMPORTANT - PLEASE READ

You may be aware of the PLOS Data Policy, which requires that all data be made available without restriction: http://journals.plos.org/plosbiology/s/data-availability. For more information, please also see this editorial: http://dx.doi.org/10.1371/journal.pbio.1001797

Note that we do not require all raw data. Rather, we ask that all individual quantitative observations that underlie the data summarized in the figures and results of your paper be made available in one of the following forms:

1) Supplementary files (e.g., excel). Please ensure that all data files are uploaded as 'Supporting Information' and are invariably referred to (in the manuscript, figure legends, and the Description field when uploading your files) using the following format verbatim: S1 Data, S2 Data, etc. Multiple panels of a single or even several figures can be included as multiple sheets in one excel file that is saved using exactly the following convention: S1_Data.xlsx (using an underscore).

2) Deposition in a publicly available repository. Please also provide the accession code or a reviewer link so that we may view your data before publication.

Regardless of the method selected, please ensure that you provide the individual numerical values that underlie the summary data displayed in the following figure panels as they are essential for readers to assess your analysis and to reproduce it:

Fig. 1A-C, E, G, I, K; Fig. 2A-D; Fig. 3F, H, J; Fig. 4H, I; Fig. 5B, D, F, G, M, O, I, K; Fig. S1A-C; Fig. S2D, F, H, J; Fig. S3B, D, F, G, I, K; Fig. S4A, B; Fig. S5A-C; Fig. S6A-D; Fig. S9F, H and Fig. S10B, D

NOTE: the numerical data provided should include all replicates AND the way in which the plotted mean and errors were derived (it should not present only the mean/average values).

Please also ensure that figure legends in your manuscript include information on WHERE THE UNDERLYING DATA CAN BE FOUND, and ensure your supplemental data file/s has a legend.

Please ensure that your Data Statement in the submission system accurately describes where your data can be found.

------------------------------------------------------------------------

CODE POLICY

Per journal policy, if you have generated any custom code during the course of this investigation, please make it available without restrictions. Please ensure that the code is sufficiently well documented and reusable, and that your Data Statement in the Editorial Manager submission system accurately describes where your code can be found. More information on our Code Policy, what and how to share can be found here: https://journals.plos.org/plosbiology/s/code-availability

Please note that we cannot accept sole deposition of code in GitHub, as this could be changed after publication. However, you can archive this version of your publicly available GitHub code to Zenodo. Once you do this, it will generate a DOI number, which you will need to provide in the Data Accessibility Statement (you are welcome to also provide the GitHub access information). See the process for doing this here: https://docs.github.com/en/repositories/archiving-a-github-repository/referencing-and-citing-content

-------------------------------------------------------------------------

BLOT AND GEL REPORTING REQUIREMENTS:

We require the original, uncropped and minimally adjusted images supporting all blot and gel results reported in an article's figures or Supporting Information files. We will require these files before a manuscript can be accepted so please prepare and upload them now. Please carefully read our guidelines for how to prepare and upload this data: https://journals.plos.org/plosbiology/s/figures#loc-blot-and-gel-reporting-requirements

Please provide the raw gels shown in the following figures: Fig. 3E, K; Fig. 4B; Fig. 5A, C, N; Fig. S1G; Fig. S9C, D and Fig. S10A, C

------------------------------------------------------------------------

DATA NOT SHOWN?

- Please note that per journal policy, we do not allow the mention of "data not shown", "personal communication", "manuscript in preparation" or other references to data that is not publicly available or contained within this manuscript. Please either remove mention of these data or provide figures presenting the results and the data underlying the figure(s).

------------------------------------------------------------------------

Reviewers' comments

Rev. 1:

In the revised manuscript from Doganli et al, the authors explore the requirement of TAK1 and TAB2, and other ciliary components in heart development. Human exome sequencing data implicated TAK1 and TAB2 as a risk factor in syndromic congenital heart defects. To model mutations in TAK1 and TAB2 in vivo, the authors generated zebrafish mutants for tak1 and tab2, which exhibited cardiac trabeculation defects, as well as craniofacial and limb defects, potentially reflecting FMD and CSCF syndromes found with human mutations. Pharmacological inhibition of Tak1 generated similar defects as the tak1 mutants. Bulk RNA sequencing of tak1 mutant hearts showed that loss of tak1 had a decrease in expression of transcription factors that promote heart development, sarcomeric proteins, and cardiac ECM genes. The authors utilized in vitro cell culture systems to show that TAK1, TAB2, and PKC-Cα localize to the primary cilium of differentiating cardiomyocytes. Human mutant versions of TAK1 do not localize to the base of cilia. Inhibition of TAK1 and TAB2 via CRISPR/Cas9 generated mutant lines and pharmacological inhibition of TAK1 blocked cardiomyocyte differentiation in vitro. TGFB addition is able to stimulate TAK1 phosphorylation and JNK1/2 phosphorylation at the base of cilia is inhibited via pharmacological inhibition of TAK1 in vitro. The authors conclude that their research provides insights into the regulation of TAK1 within the cilia and sheds new light on its role in cardiomyocyte development and potentially the causes of syndromic congenital heart defects.

The revised manuscript is much improved over the previous submission. It is appreciated that the authors we responsive to the majority of the previous critiques. The interpretations are more in line with their presented data. The actual mechanism underlying the trabeculation and valve defects in the zebrafish is not explained nor are the differences between the in vitro and in vivo data. However, the authors address the latter issue in the discussion. Overall, there is a significant amount of data supporting the molecular mechanism that is proposed in this improved manuscript, which may support a novel mechanisms of action within the cilia underlying syndromic congenital heart defects.

The statistical analysis used appears appropriate. Comments regarding supplementary data are below. Accession numbers for data have been supplied.

I have a some remaining minor suggestions that the authors may want to consider with respect to their final manuscript.

Minor issues:

1. On page 12, the sentence with "displayed no defect" is awkward and should be edited.

2. The increased heart rate in the mutants seems statistically different, but is this biologically meaningful? The mean for wt and tak1 mutants is 140 bpm vs 150 bpm. It seems to receive greater emphasis than needed in the manuscript. What this means is nor is its relationship to trabeculation clear and the cause of the tachycardia, like effects of Tak1 on pacemaker cells, are not examined.

3. Some Figures are presented out of order, in particular Fig. S4A.

4. Fig. S4A is difficult to interpret due to the angles used for imaging. It is hard to see the bulbus is actually reduced in size in the mutants. Presenting the different imagining channels may make this point clear.

5. The "normalized atrial area" as in Fig. S5A and S5B is still not a good way to examine chamber size compared to the quantification the authors have not performed. It could have a lot of different interpretations that are not really covered. This analysis should be qualified within the text.

6. On page 16, the "data not shown" might be good to show in the supplemental data, as the staining in the Fig. 3A is relatively minimal.

7. In Fig. 4F, the insets of Ac-tub/DAPI/TAB2 are pixelated.

8. It might be good to show to examine pSmad2/3 in the tak1 and tab2 mutant hearts, like they did with the RPE-1 cells.

9. With regard to interpretations of the RNA-seq data, the associated with terms related to fibroblasts likely reflects changes in epicardial cells, which is 3rd on the list in Fig. 2D, or the bulbus. The zebrafish heart does not have fibroblasts at that stage.

Rev. 3:

The authors have adressed most of my concerns but not all.

- Based on the images in Fig.3A and without a cardiomyocyte specific marker, they cannot conclude that Tak1 localizes to primary cilia in cardiomyocytes in zebrafish. The cilium can be seen inside the heart but whether the cilium is on an endocardial or myocardial cell in not evident. Therefore, they should adjust their conclusion that TAK1 localizes to cilia in cardiomyocytes in zebrafish.

- In addition, the TAK1 localisation in gastruloids also indicates that it is not exclusive to nkx2.5+ cells but also present in other (nkx2.5-) cells. Evidence that TAK1 operates in cardiomyocytes is very weak and cardiac phenotypes observed can also be explained by effects on other (non-myocardial) cells. In addition, TAK1 has very different effects on cardiomyocytes in vitro versus in vivo experiments. Therefore they need to tune down the suggestion that TAK1 has a specific role in cardiomyocytes and cardiomyogenesis and rather refer to a role of TAK1 in cardiac development.

- Methods section: Please include the temperature at which cardiac function was assesed as heart rate and cardiac output are influenced by temperture.

Rev. 4:

The authors have addressed my major concerns. I do not have any further comments.

# Author comment: Author response to Decision Letter 2

**DOI:** 10.1371/journal.pbio.3003902.r005

23 Jun 2026

Attachment Submitted filename: Response to reviewers-R2.docx

# Editor report: Decision Letter 2

**DOI:** 10.1371/journal.pbio.3003902.r006

**Authors:** Ines Alvarez-Garcia

29 Jun 2026

Dear Dr Larsen,

Thank you for the submission of your revised Research Article entitled "TAK1 operates at the primary cilium in non-canonical TGFB/BMP signaling to control heart development" for publication in PLOS Biology. On behalf of my colleagues and the Academic Editor, Mary Mullins, I am delighted to let you know that we can in principle accept your manuscript for publication, provided you address any remaining formatting and reporting issues. These will be detailed in an email you should receive within 2-3 business days from our colleagues in the journal operations team; no action is required from you until then. Please note that we will not be able to formally accept your manuscript and schedule it for publication until you have completed any requested changes.

Please take a minute to log into Editorial Manager at http://www.editorialmanager.com/pbiology/, click the "Update My Information" link at the top of the page, and update your user information to ensure an efficient production process.

PRESS

We frequently collaborate with press offices. If your institution or institutions have a press office, please notify them about your upcoming paper at this point, to enable them to help maximise its impact. If the press office is planning to promote your findings, we would be grateful if they could coordinate with biologypress@plos.org. If you have previously opted in to the early version process, we ask that you notify us immediately of any press plans so that we may opt out on your behalf.

We also ask that you take this opportunity to read our Embargo Policy regarding the discussion, promotion and media coverage of work that is yet to be published by PLOS. As your manuscript is not yet published, it is bound by the conditions of our Embargo Policy. Please be aware that this policy is in place both to ensure that any press coverage of your article is fully substantiated and to provide a direct link between such coverage and the published work. For full details of our Embargo Policy, please visit http://www.plos.org/about/media-inquiries/embargo-policy/.

Many congratulations and thanks again for choosing PLOS Biology for publication and supporting Open Access publishing. We look forward to publishing your study.

Sincerely,

Ines

--

Ines Alvarez-Garcia, PhD

Senior Editor

PLOS Biology

ialvarez-garcia@plos.org
